# Safety, Immunogenicity, Efficacy and Effectiveness of Inactivated Influenza Vaccines in Healthy Pregnant Women and Children Under 5 Years: An Evidence-Based Clinical Review

**DOI:** 10.3389/fimmu.2021.744774

**Published:** 2021-10-06

**Authors:** Amit Bansal, Mai-Chi Trieu, Kristin G. I. Mohn, Rebecca Jane Cox

**Affiliations:** ^1^ The Influenza Centre, Department of Clinical Sciences, Faculty of Medicine, University of Bergen, Bergen, Norway; ^2^ Department of Medicine, Haukeland University Hospital, Bergen, Norway; ^3^ Department of Microbiology, Haukeland University Hospital, Helse Bergen, Bergen, Norway

**Keywords:** inactivated influenza vaccine (IIV), safety, immunogenicity, vaccine efficacy, vaccine effectiveness (VE), young children, pregnant women

## Abstract

Annual influenza vaccination is often recommended for pregnant women and young children to reduce the risk of severe influenza. However, most studies investigating the safety, immunogenicity, and efficacy or effectiveness of influenza vaccines are conducted in healthy adults. In this evidence-based clinical review, we provide an update on the safety profile, immunogenicity, and efficacy/effectiveness of inactivated influenza vaccines (IIVs) in healthy pregnant women and children <5 years old. Six electronic databases were searched until May 27, 2021. We identified 3,731 articles, of which 93 met the eligibility criteria and were included. The IIVs were generally well tolerated in pregnant women and young children, with low frequencies of adverse events following IIV administration; however, continuous vaccine safety monitoring systems are necessary to detect rare adverse events. IIVs generated good antibody responses, and the seroprotection rates after IIVs were moderate to high in pregnant women (range = 65%–96%) and young children (range = 50%–100%), varying between the different influenza types/subtypes and seasons. Studies show vaccine efficacy/effectiveness values of 50%–70% in pregnant women and 20%–90% in young children against lab-confirmed influenza, although the efficacy/effectiveness depended on the study design, host factors, vaccine type, manufacturing practices, and the antigenic match/mismatch between the influenza vaccine strains and the circulating strains. Current evidence suggests that the benefits of IIVs far outweigh the potential risks and that IIVs should be recommended for pregnant women and young children.

## Highlights

Inactivated influenza (flu) vaccine is recommended by the WHO for all pregnant women and children aged 6 months to 5 years. Flu is more likely to cause severe illness in pregnant women, and young children are the main transmitters of the virus. Vaccination against influenza lowers the risk of severe complications from flu during pregnancy, infancy, and early childhood. The benefits of influenza vaccination to the mother and child outweigh the potential risks.

## Introduction

Influenza occurs in epidemics of variable impacts every year. Among the four types of viruses—A, B, C, and D—only influenza A and B viruses cause seasonal epidemics. Influenza A virus subtypes are divided by the two major viral membrane glycoproteins, namely, hemagglutinin (HA) and neuraminidase (NA), and influenza B viruses have two lineages (Victoria and Yamagata). Minor changes by point mutations in the RNA gene segments that code for the HA or NA of influenza A and B viruses (antigenic drift) may result in seasonal influenza epidemics of variable intensities and severities (1). Major changes in human influenza A viruses associated with the acquisition of novel HA with or without novel NA proteins occur through genetic reassortment with animal influenza viruses or, potentially, direct zoonotic transmission and are referred to as antigenic shift. Such events led to the emergence of novel viruses, which caused pandemics in 1918 (H1N1), 1957 (H2N2), 1968 (H3N2), and 2009 (H1N1pdm09) (1–3). Zoonotic influenza viruses infect humans through direct transmission, such as avian flu, but only are considered pandemic if they can spread from human to human. The continuous evolution of influenza viruses influences the severity of influenza seasons and poses a continuous threat to human health.

The influenza virus infects all age groups. However, pregnant women and young children are especially at high risk of influenza complications, resulting in serious illness, increased hospitalizations, and mortality (4, 5). The laboratory-confirmed influenza (LCI) hospitalization and mortality rates were 0.4–77 cases (6–9) and 0.3–6.9 cases (6, 8–10) per 100,000 pregnancies, respectively, during the 2009 pandemic. The annual global influenza attack rates are estimated to be higher in children (20%–30%) than that in adults (5%–10%) (11). Influenza accounts for 7%–13% of acute lower respiratory infections in children <5 years globally (5, 12). Unlike in temperate zones where a clear influenza activity peak occurs in the winter, influenza activity can occur year-round in tropical or subtropical countries, with a peak in the monsoon season. In Bangladesh, the influenza incidence among children <5 years old ranged from 6.3 episodes/1,000 child-years in January to 258.3 episodes/1,000 child-years in May during the 2004–2007 seasons (13), whereas in Finland (14), the influenza attack rates were 175 to 179 per 1,000 young children in the 2000–2002 seasons. The risk of influenza-related serious illness or hospitalizations (15–18) and all-cause mortality (19) is higher in young than that in older children, especially in children under 6 months of age (20, 21). The most common complications of pediatric influenza are pharyngitis (range = 31%–58% of LCI), acute otitis media (range = 0%–41%), and febrile seizures or convulsions (range = 0%–45%) (22). The majority of influenza-related deaths in young children occur in developing countries (5, 12). Furthermore, young children play an important role in the community spread of influenza (22).

The role of natural influenza infection in the protection against subsequent infection is not well studied, especially in pregnant women and young children, although most people will have been infected with influenza during early childhood. Influenza infection induces a multifaceted and long-lived immunity, whereas vaccination induces a more specific and short-lived immunity [review in (23)]. However, influenza viruses can escape the infection-established immunity and subsequent natural reinfection occurs (24). A human challenge study suggested that sequential infection with identical influenza A viruses can also occur (25). Therefore, influenza vaccination remains the best preventive method against influenza infection and its related complications (15, 16). Two types of influenza vaccines are available: inactivated influenza vaccines (IIVs) and live attenuated influenza vaccines (LAIVs). LAIVs are used in children >2 years (in Europe and the USA), but are contraindicated in pregnant women, children <2 years old, and immunosuppressed individuals, while IIVs are used in individuals >6 months old and in pregnant women. Influenza vaccine purity has been greatly improved over the last 60 years; however, the whole-virus IIVs were found to be significantly reactive in young children (26). This resulted in the development of split-virus IIVs in which reactogenicity was reduced by detergent treatment of the virus, which, in some cases, is further purified into surface antigen subunits (26).

Twice a year, the World Health Organization (WHO) makes recommendations on the composition of the influenza vaccine, currently including three or four influenza strains (A/H1N1, A/H3N2, and one or two influenza B strains) that are predicted to circulate in the upcoming seasons (trivalent or quadrivalent IIVs, TIV or QIV). Several countries recommend QIV for pregnant women and young children due to the high burden of influenza B illness and the potential of mismatch between the circulating influenza B viruses and the vaccine strains in TIV (27, 28). Pregnant women require one annual IIV dose, while children 6 months to 8 years old require two doses as a prime–boost regime to ensure adequate seroprotection against influenza (29, 30). Thereafter, only one annual dose is required. Comparing vaccine immunogenicity and vaccine effectiveness (VE) estimates between the different vaccines is challenging due to several factors, such as the IIV type (whole-virus, virosome, split-virus, or subunit), with/without adjuvant, and the manufacturing processes (eggs, cell culture, or recombinant protein) (31). Other confounding factors (31) are the vaccinee’s age, preexisting immunity, comorbidities, and antigenic match/mismatch between the vaccine strains and circulating viruses.

IIVs have been used for over 60 years and given to hundreds of millions of people, providing good safety and immunogenicity records (32–39). However, limited evidence exists from randomized controlled trials (RCTs) on the efficacy of maternal influenza vaccine against serious illnesses (4). Furthermore, the majority of VE studies originate from high-income countries (4), with low- and middle-income countries underrepresented, especially for young children. Here, we provide an evidence-based clinical review on the safety, immunogenicity, and efficacy/effectiveness of IIVs in healthy pregnant women and children <5 years old with an emphasis on data from low- and middle-income countries.

## Search Strategy

**Figure 1:**
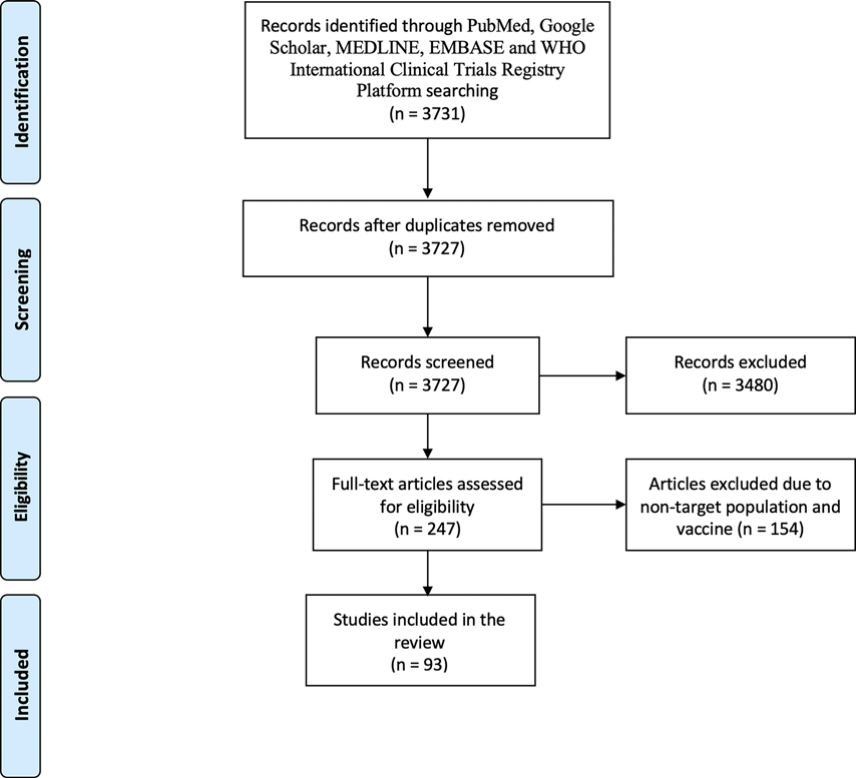
Flowchart of the included studies. We accessed 3,727 studies (titles and abstracts) following deletion of duplicates (*n* = 4). The literature search strategy included the following keywords: “influenza,” “maternal influenza vaccination,” “humans,” “pregnant women,” “young children,” “safety,” “adverse event/effect,” “immunogenicity,” “vaccine efficacy,” “vaccine effectiveness,” and “inactivated influenza vaccines” [and the Boolean operators (OR and AND)]. Eligible studies met the following inclusion criteria: 1) published from database inception to May 27, 2021, and 2) evaluated the safety profiles, immunogenicity, and effectiveness of inactivated influenza vaccines (IIVs) in healthy pregnant women and children <5 years old. Studies were excluded based on the title and abstract; non-peer-reviewed papers were not included. Large-scale studies were included only if the results were stratified for the target population. Studies in non-English language were also accessed. Most of the randomized controlled trials in pregnant women included in this review were conducted in low- and middle-income countries, while observation studies were conducted in high-income countries. Out of 93 suitable studies, the majority evaluated seasonal Northern Hemisphere IIVs, even when a study was conducted in the Southern Hemisphere, and 12 studies evaluated H1N1-pdm09 infection and/or vaccination. Of these 93 studies, 36 were on IIV safety, 10 on immunogenicity in pregnant women (two studies assessed both safety and immunogenicity), 16 on immunogenicity in young children (10 studies assessed both safety and immunogenicity), 17 studies for IIV effectiveness in pregnant women (three assessed both immunogenicity and effectiveness and one assessed both safety and effectiveness), and 33 studies for IIV effectiveness in young children (one on both safety and effectiveness and two on both immunogenicity and effectiveness).

The electronic databases PubMed, Google Scholar, MEDLINE, Embase, WHO International Clinical Trials Registry Platform (ICTRP), and UpToDate were searched using these keywords: “influenza,” “maternal influenza vaccination,” “humans,” “pregnant women,” “young children,” “safety,” “adverse event/effect,” “immunogenicity,” “vaccine effectiveness,” and “inactivated influenza vaccines.” Eligible studies met the following inclusion criteria: 1) published from inception to May 27, 2021, and 2) evaluated the safety profile, immunogenicity, or effectiveness of IIVs in healthy pregnant women or children <5 years old. Studies were excluded based on the title and abstract. Articles resulting from these searches and relevant references cited in those articles were reviewed. We accessed 3,731 studies. After screening, 93 studies were included in this review (**Figure 1**), of which 36 studies were on IIV safety, 10 and 16 on immunogenicity, and 17 and 33 were on the efficacy/effectiveness of IIVs in pregnant women and children, respectively. Most of the randomized controlled trials in pregnant women included in this review were conducted in low- and middle-income countries, while observation studies were conducted in high-income countries.

## Tolerability of Inactivated Influenza Vaccines in Pregnant Women and Children Under 5 Years Old

Out of 93 studies, 36 were on the safety of IIVs in pregnant women and young children. Numerous studies have found that IIVs are safe for pregnant women (32, 33, 36, 39–49) and children <5 years old (34–37, 40–43, 45–47, 50, 51). In one study, no clinically significant difference was found in birth weight or gestational age at birth between infants of mothers who were vaccinated with IIV during any trimester of pregnancy and infants of unvaccinated mothers (32). There was no increased risk of either maternal complications or unwanted fetal outcomes after vaccination (33). In children, the most common adverse events after IIV were transient pain and irritability on the injection site, but no reaction persisted for more than 4 days (35). The majority of seasonal IIV studies report that adverse events requiring medical care are uncommon, and few children (up to 5.4%) experience febrile illness after IIV (36, 52–57), while some studies (34, 58–61) report transient fever in up to 18% of children. TIV has been temporally associated with hives (57), Henoch–Schönlein purpura (62), and pharyngitis (63) in children, but no causal evidence exists (64). Overall, the safety profiles are similar for the split-virion QIV and TIV in children 6–35 months old, except for more reactions on the injection site with QIV than with placebo or TIV (37).

There have been concerns over severe adverse events after IIV, such as narcolepsy and Guillain–Barré syndrome, but no indication of causality was found, although a weak correlation between IIV and narcolepsy, Guillain-Barré syndrome, or severe febrile illness was suggested in children (33–37, 65). A pre-registration pediatrics study (66) in the USA comparing two TIV formulations (TIV by CSL and Fluzone by Sanofi Pasteur Inc.) in 6- to 36-month-olds reported higher rates of fever (37.1% vs. 13.6%), severe fever (2.6% or 0%), irritability (58.5% or 37.3%), and loss of appetite (31.9% or 19.7%) after the first dose of the formulation approved for use in adults only, but the rates were lowered after the second dose. In 2010, there was a suspension of pediatric IIV use in Australia that lasted several years because Southern Hemisphere TIVs (manufactured by CSL Biotherapies, King of Prussia, PA, USA) were associated with severe febrile illness in children, but this was not found with the other IIVs (67). However, concomitant pneumococcal vaccination may further enhance the risk of febrile seizures in young children (68). Recent large-scale Australian cohort studies (69, 70) have found low rates of febrile illnesses in young children and pregnant women following QIV. Furthermore, IIV decreased the antepartum hospitalization risk of maternal influenza-like illness (ILI) by 39% (70). IIVs are not normally adjuvanted, but three main adjuvants have been used in pandemic IIVs (38): MF59, AS03, and AF03. Only MF59 is currently used in seasonal vaccines. The MF59-adjuvanted influenza vaccine was well tolerated in young children and induced greater, longer-lasting, and broader immune responses compared to the non-adjuvanted split vaccine (58, 71); however, transient and mild solicited reactions were more frequent after MF59-adjuvanted IIVs. Notably, the AS03-adjuvanted 2009 pandemic IIVs are no longer licensed for use in children <20 years old in Europe due to the association of the AS03-adjuvanted A/H1N1pdm09 vaccine with onset of narcolepsy in children in Scandinavia (36, 72). The whole-virus IIVs are also not used in Europe and North America due to safety concerns of increased reactogenicity (23). The prenatal 2009 pandemic A/H1N1pdm09 vaccination (with/without AS03) was weakly associated with increased risk of asthma and decreased rates of gastrointestinal infections in young children, which were attributed to confounding factors (50). A Danish retrospective cohort study (*n* = 61,359) concluded that the antenatal monovalent AS03-adjuvanted split-virion A/H1N1pdm09 IIVs were safe without any augmented early childhood morbidity risk (40). The adverse events of special interest for the AS03-A/H1N1pdm09 IIVs are generally rare, and no unexpected events during pregnancy were reported, but young children had higher reporting rates of adverse events (73). The risk of Guillain–Barré syndrome after IIV, particularly influenza A/H1N1pdm09, is very low (74–76) and lower than that with influenza infection (77). Furthermore, no significant links were found between H1N1pdm IIV and infectious diseases (e.g., pulmonary infections and otitis media), cancers, sensory disorders, use of hospital services, pediatric chronic diseases, and mortality (50).

This review may help physicians, pregnant women, and parents make informed choices about influenza immunization. IIVs are safe and well tolerated (36, 38, 40, 50, 51, 65) for pregnant women and children <5 years, but continued monitoring of adverse events is necessary to detect infrequent events.

## Criteria of IIV Immunogenicity

The criteria of the European Committee for Medicinal Products for Human Use (CHMP) have been widely used to evaluate vaccine immunogenicity using the hemagglutination inhibition (HI) assay. Serum HI titer ≥40 is associated with >50% reduction in influenza infection or disease and is considered as a surrogate correlate of protection (COP) (78). However, this protective titer was established in healthy adults and not confirmed in children, with some experts recommending HI titres >110 (79) and others >160 (80). In adult vaccinees, as a part of registration requirements, three criteria (81) should be met, namely, >40% seroconversions or significant increase in HI titers, >2.5-fold increase in geometric mean HI titers (GMTs), and >70% seroprotection (achieving HI titer ≥40), for pandemic IIV and at least one of these criteria for seasonal IIV. HI and microneutralization (MN) titers are significantly correlated with each other; however, no MN titer level has been defined as a COP. In children, MN is the most sensitive for protection against seasonal A/H3N2 (82). Since 2017, new CHMP guidelines have recommended the measurement of neutralizing antibodies in addition to the HI titer and encouraged the assessment of broader immune response anti-neuraminidase antibodies, antibody kinetics, and cell-mediated immunity (83).

### Immunogenicity of IIV in Pregnant Women and Their Newborns

IIV induces strong humoral responses in pregnant women (84–88). After IIV administration, a 6- to 10-fold increase in GMTs (85, 88) and >72% seroconversions (85) were observed in pregnant women. A US cohort study (87) found that seasonal IIV led to 65%–95% and 75%–98% seroprotection rates for influenza A/H3N2 and A/H1N1, respectively, in pregnant women, with no significant difference by trimester or postpartum. However, the seroconversion rates were highest in the late third trimester and the postpartum period and lower in women with obesity (87). Higher pre-vaccination antibody levels and prior influenza vaccination were both linked to reduced odds of seroconversion rates, suggesting the antibody ceiling effect. Albeit a small study (*n* = 56), the seroprotection rates after TIV were largely comparable between pregnant and non-pregnant women for A/H1N1 (89% *vs.* 85%), A/H3N2 (81% *vs.* 93%), and B (83%% *vs.* 100%) during the 2011–2012 influenza season in the USA (89). Among pregnant TIV recipients in South Africa, Madhi et al. (84, 85) found 93%, 78%, and 96% seroprotection rates against A/H1N1pdm09, A/H3N2, and B/Victoria, respectively, and a 54% corresponding total vaccine efficacy against confirmed influenza. TIV induced significant enhancements of both the MN and HI titers against the three vaccine strains, and the MN titers were two to threefold higher than the HI titers, except against B/Victoria (90). Pregnant women vaccinated with QIV had similar safety and enhancement of GMTs for the strains included in TIV, but the GMTs were significantly higher for the second B strain (49). A Norwegian cohort study found durable antibody response after the 2009 AS03-adjuvanted monovalent pandemic IIV in pregnant women since the estimated waning of antibodies was slower in vaccinated pregnant women than that in ILI cases (HI titer half-life of 260 *vs.* 192 days) (39).

Importantly, infants born to vaccinated pregnant women received the complementary benefit of vertically transferred immunity against influenza (84, 85), a potentially cost-effective strategy. An RCT (*n* = 322) in South Africa (86) found that the percentages of infants with influenza-specific antibodies born to TIV-recipient mothers were significantly higher than those in saline placebo-recipient mothers (HI titers ≥40 at birth 78% *vs.* 34% against A/H1N1pdm09, 57% *vs.* 17% against A/H3N2, and 81% *vs.* 42% against B/Victoria, respectively). Yet, the percentage (86) of infants with seroprotective titers decreased from birth to 6 months (91). Passive immunity through placental transfer of maternal immunoglobulin G (IgG) antibodies to the fetus and IgA antibodies through breastfeeding is important to protect newborns against influenza infection (92), although transplacental antibody transfer seems to be the key mechanism in protecting newborns against influenza rather than through breast milk (93). Transplacental antibody transfer (94), primarily IgG, typically starts from 17 weeks of gestation and peaks at 37–41 weeks, with enhanced neonatal Fc receptor expression. Antibody decay occurs during the first 2–3 months (93, 95) in newborns. IIV immunization later in the trimester led to significantly higher seroprotection rates 2–3 days after delivery (effect sizes increase between the first and the third trimester), but no significant difference in the cord blood seroprotection rates was found between women vaccinated in the second or the third trimester (95). However, antibodies may wane faster in women vaccinated later in pregnancy, with some studies estimating antibody half-life of 7 weeks (95). Notably, there were fewer studies with the first trimester IIV immunogenicity results, where vaccination is often not recommended due to worries about temporal association with spontaneous abortion. The seroprotection and seroconversion rates at delivery were mostly high in pregnant women regardless of the vaccination timing (96).

Current limited evidence suggests that the IIV elicits similar good antibody responses in pregnant and non-pregnant women, and the antibodies can passively be transferred to their newborns. Although the vaccination timing may influence the antibody levels in pregnant women and their newborns, cumulative transfer of antibodies suggests the need for early IIV immunization of pregnant women (93).

### Immunogenicity of IIV in Children Under 5 Years

A Canadian dose–response RCT (34) in 6- to 23-month-old children (*n* = 252) of the 2008–2009 Northern Hemisphere TIV found >85% seroprotection rates for all three vaccine strains in children aged 12–23 months without significant difference by dose (full *vs.* half dose), while the full dose induced higher antibody responses against all three vaccine strains without increasing reactogenicity in unprimed children aged 6–11 months. These results differ from an RCT (97) conducted in the USA in children 6–35 months of age, where increasing the antigen content of the 2010–2012 Northern Hemisphere TIV did not significantly increase the antibody responses to any of the three vaccine strains, except for the primed group who had been previously infected/vaccinated with A/H1N1 (as well as a subgroup analysis with infants only). Another Canadian RCT (35) evaluated the immunogenicity of the 2008–2009 Northern Hemisphere TIV in 6- to 35-month-old children (*n* = 374), reporting that two of the three serological criteria (>40% seroconversion rate and >2.5-fold increase in GMTs) were met for all vaccine strains and for both doses (full *vs.* half dose) in all TIV groups. Furthermore, antibody responses were significantly higher in children aged 24–35 months than those in 6–23 months. Similarly, higher antibody responses to TIV were reported with increasing age and after the second dose in healthy influenza-naive children 6–23 months old in the USA, albeit TIV was immunogenic in children 6–11 months old (98). Several RCTs in 2002–2003 (USA) (54, 55) and 2006–2009 (Europe) (58–60) found seroprotection rates ranging from 70% to 100% in children <5 years old following the Northern Hemisphere TIV. IIV is licensed for children from 6 months of age, and there are few studies evaluating the immunogenicity of IIV in children less than 12 months old. One study in infants reported >90% seroprotection rate in TIV recipients for at least one virus and 49.6% for two strains compared to 16.4% and 0.9%, respectively, in placebo recipients (57). Pilot data suggested that 6- to 12-week-old infants have significantly lower (99) antibody responses to TIV than do 6-month-old babies, with post-vaccination seroprotection rates of 46% *vs.* 69% against A/New Caledonia (H1N1), 59% *vs.* 79% against A/Wyoming (H3N2), and 5% *vs.* 22% against B/Jiangsu. In general, studies reported good immunogenicity profiles in children <5 years old (34, 35, 54–60, 66, 98, 99).

A multicenter phase III RCT (100) evaluated the immunogenicity of a split-virion QIV (2013–2014 Northern Hemisphere) in children 3–8 years old in Poland, Finland, Mexico, and Taiwan (*n* = 1,242) and found that the post-vaccination GMTs were augmented by more than sixfold for all vaccine strains (6.86 for A/H1N1, 7.49 for A/H3N2, 17.1 for B/Victoria, and 25.3 for B/Yamagata), regardless of the comparatively high baseline HI titers. The immunogenicity profile of QIV was comparable to that of TIV, with superiority for the second B strain (100, 101). More recently, a phase III RCT (37) evaluated the safety and efficacy of a split-virion QIV in healthy, previously unvaccinated children aged 6–35 months (*n* = 5,806) in Latin America, Asia, Africa, and Europe during the Northern Hemisphere and Southern Hemisphere 2014 and 2015 influenza seasons. The study found that most children were seronegative at baseline for each of the four vaccine strains, except only 50% of children from Asia were seronegative during the 2014 Southern Hemisphere season. After two doses of QIV, the seroconversion rates were >87% at day 56 for all four vaccine strains, and only <2% of participants remained seronegative (37). Moreover, the neutralizing antibody responses after IIV have not been extensively analyzed in children. One study found neutralizing antibodies against all homologous and heterologous H1 and H3 strains tested in all age groups, including children 2–8 years old receiving the 2009–2010 seasonal IIVs (102). Children aged 2–8 years also had higher seroprotection and seroconversion rates to homologous and heterologous strains compared to adults.

In conclusion, IIV elicits good HI antibody responses in children <5 years old, although there are no established HI protective titers for children and many studies rely on the adult criteria for HI titers (≥40 and a fourfold rise) when documenting seroprotection rates. Work is needed to evaluate protective HI titers and neutralizing antibodies in young children in order to better determine vaccine immunogenicity in this age group.

## Vaccine Efficacy/Effectiveness of IIVs in Pregnant Women and Children Under 5 Years

Vaccine efficacy and vaccine effectiveness (VE) measure the proportionate reduction in cases among vaccinated people under ideal (i.e., a randomized controlled trial) or typical field conditions, respectively. VE is now often assessed using a test-negative design in which patients with an ILI are tested for influenza. To estimate VE, vaccine coverage is compared between those testing positive *versus* those testing negative. Due to limited VE data on IIVs in pregnant women and children, we have included studies with both vaccine efficacy and VE below.

### Vaccine Efficacy/Effectiveness and Health Benefits of IIVs in Pregnant Women and Their Newborns

In an RCT in South Africa, Madhi et al. (4, 85) demonstrated that TIV partly protected pregnant women and their newborns, with vaccine efficacy values of 50% and 49%, respectively, against LCI during a 6-month postpartum follow-up period. Nunes et al. (86) conducted a secondary analyses of the data (85) and found that the vaccine efficacy against LCI decreased with age: 86% (95%CI = 38%–98%) efficacy in babies ≤8 weeks old, 25% (95%CI = −68% to 68%) in 8–16 weeks old, and 30% (95%CI = −155% to 83%) in 16–24 weeks old. Furthermore, the efficacy in newborns ranged from 30% in the Nepalese to 63% in Bangladeshi RCTs (103), confirming that maternal IgG antibodies can cross the placenta and protect newborns against influenza infection. Transplacental (and breast-milk-mediated) antibody transfer is an important means of protecting infants <6 months who are at high risk of hospitalization due to influenza (104) and IIV is not approved.

Observational data (36, 105, 106) showed that IIVs were 44%–65% effective against influenza among pregnant women, but the confidence intervals are wide, suggesting imprecise estimates (**Table 1**). Limited data exist on VE against severe influenza (requiring hospitalization) among pregnant women. A retrospective cohort study (118) in the USA from the 2005–2006 to 2013–2014 seasons concluded that infants born to vaccinated mothers, including those with comorbidities, had 70% risk reductions of LCI and 81% in influenza hospitalizations in the first 6 months of life. Similar results were reported in England during the 2013–2014 seasons, where antenatal IIVs were 71% effective (95%CI = 24%–89%) in preventing influenza infection and 64% effective (95%CI = 6%–86%) in preventing influenza hospitalization (113). A matched case–control study (115) in the USA reported that seasonal IIV immunization in pregnant women was 91.5% effective in preventing newborn hospitalization due to influenza. However, the selection bias might have overstated the VE estimates.

**Table 1:** Vaccine efficacy and effectiveness of influenza vaccination in healthy pregnant women.

Vaccine	No. of participants (intervention and control groups)	Outcomes in mothers	Effect sizes in IIV-vaccinated mothers (95%CI)	Effect sizes and AR of symptomatic LCI in newborns in VA and CA (95%CI)	First author, year (region, quality)
**RCTs**					
Seasonal TIV 2004 SH and pneumococcal	340 (172 in TIV and 168 in pneumococcal)	VE against respiratory illness with: a) any fever; b) temperature >38°C; c) diarrheal disease; and d) clinic visit	a) 35.8 (3.7–57.2); b) 43.1 (−9.0 to 70.3); c) 19.3 (−24.6 to 47.8); d) 24.9 (−43.9 to 60.8)	VE: 63% (5–85) and 0.7% *vs.* 1.8%	Zaman, 2008 (4, 41, 107) (Bangladesh, high) (107)
Seasonal split-virion TIV 2011 and 2012 SH	2,116 (1,062 in TIV and 1,054 in saline placebo)	Vaccine efficacy against LCI AR of symptomatic LCI	50.4% (14.5–71.2) VA *vs.* CA: 1.8% *vs.* 3.6%	Vaccine efficacy: 48.8% (11.6–70.4) and 1.9% *vs.* 3.6%	Madhi, 2014 (4, 85) (South Africa, high) (107)
Seasonal TIV (2011 NH, 2012 N and 2012 SH)	4,193 (2,108 in TIV and 2,085 in meningococcal vaccine)	Vaccine Efficacy AR of symptomatic LCI	70.3% (42.2–85.8) VA *vs.* CA: 0.5% *vs.* 1.9%	Vaccine efficacy: 33.1% (3.7–53.9) and 2.5% *vs.* 3.8%	Tapia, 2016 (4, 108) (Mali, high) (107)
Seasonal TIV (2010–2012 NH and 2011–2013 SH)	3,693 (1,847 in TIV and 1,846 in saline placebo)	LCI (pregnancy) LCI (postpartum)	0.77 (0.42–1.43) 0.59 (0.28–1.23)	Vaccine efficacy: 30% (5–48) and 4.1% *vs.* 5.8%	Steinhoff, 2017 (4, 109) (Nepal, high) (107)
Seasonal TIV 2011–2012 NH, 2012–2013 SH	3,693 (1,847 in TIV and 1,846 in saline placebo)	a) Flu IRR (pregnancy) and b) flu IRR (postpartum) for immunization at 17–25 and 26–34 weeks gestation	a) 0.62 (0.30–1.31) and 1.32 (0.42–4.14); b) 0.62 (0.25–1.54) and 0.56 (0.16–1.90)	Infant influenza IRR: 0.73 (0.51–1.05) and 0.63 (0.37–1.08)	Katz, 2018 (107, 110) (Nepal, high) (107)
**Cohort studies**					
IIV 2002–2009	1,510 hospitalized infants	Hospitalization rates for influenza-vaccinated mothers compared to those unvaccinated	45%–48% less likely	Infant LCI: 6% (*n* = 18) *vs.* 11% (*n* = 133)	Poehling 2011 (111) (USA, poor) (107)
Seasonal IIV 2002–2005 thiomersal-reduced	1,160 mother–infant pairs	NA	NA	Risk reduction of LCI in infants: 41% (7–63)	Eick 2011 (91) (USA, fair) (107)
Seasonal TIV 2012–2013 SH (four brands)	3,007 and 31,694	VE in preventing ED visits VE in preventing hospital admission	81% (31−95) 65% (3−87)	NA	Regan 2016 (106) (Australia, fair) (107)
Seasonal TIV 2010	106 and 90	NA	NA	Infant LCI: 0 (0%) *vs.* 5 (5.6%)	Sigumura 2016 (112) (Japan, poor) (107)
**Screening methods**					
Seasonal IIV 2013–2014	37 LCI infants	NA	NA	Adjusted VE for preventing influenza and related hospitalization: 71% (24–89) and 64% (6–86)	Dabrera 2014 (113) (UK, poor) (107)
Seasonal IIV 2013–2014 and 2014–2015	37 LCI infants in 2013–2014 and 81 in 2014–2015	NA	NA	Adjusted VE against influenza-related hospitalization: 66% (18–84) in 2013–2014 and 50% (11–72) in 2014–2015	Walker 2020 (114) (UK)
**Case–control studies**					
Seasonal IIV 2000–2009	205 (113 cases and 192 controls)	NA	NA	Adjusted VE against influenza-related hospitalization: 91.5% (61.7–98.1)	Benowitz 2010 (115) (USA, fair) (107)
Seasonal TIV 2010–2012 NH	292 (100 cases and 192 controls)	Adjusted VE for current TIV use	58% (14–79)	NA	Thompson^a^, 2014 (105) (USA)
Seasonal TIV2010–2017 NH	920 (460 cases and 460 controls)	VE against LCI	63.9% (29.1–81.6) (*n* = 313)	VE against LCI: 56.8% (25.0–75.1) (*n* = 460) in infants <6 months	Molgaard-Nielsen 2019 (116) (Denmark, good) (107)

IIV, Inactivated influenza vaccine; RCTs, randomized controlled trials; TIV, trivalent influenza vaccine; SH, WHO Southern Hemisphere recommended strains; NH, WHO Northern Hemisphere recommended strains; VE, vaccine effectiveness; LCI, laboratory-confirmed influenza; VA, vaccine arm; CA, control arm; AR, attack rate; NA, not available.

a Test-negative design (117) used to evaluate the effectiveness of influenza vaccines.

In a Norwegian registry-based study (119), the pandemic monovalent H1N1pdm09 IIV with the AS03 adjuvant administered during pregnancy substantially reduced the risk of an influenza diagnosis (adjusted hazard ratio = 0.30), and it may have minimized the risk of influenza-related fetal demise during the pandemic. A Japanese questionnaire-based study (120) evaluating the 2009 H1N1 pandemic VE concluded that vaccination reduced infection by 89% in pregnant women.

In addition to the value of maternal–fetal transfer of influenza-specific antibodies (45, 84, 121) following antenatal IIV, there are also complementary benefits to vaccinated pregnant women. Women who received antenatal IIV had a lower risk of preterm delivery (13%) and low-birth-weight (26%) babies compared with unvaccinated pregnant women (70, 122). Furthermore, maternal influenza immunization has been linked to a decreased risk of having low-birth-weight babies in Bangladeshi and Nepalese RCTs (103).

### Vaccine Efficacy/Effectiveness and Other Benefits of IIVs in Children Under 5 Years

Studies conducted in young children reported varying levels of vaccine efficacy/effectiveness primarily depending upon whether the circulating viruses match the vaccine strains and their preexisting immunity. Claeys et al. (123) conducted a multinational RCT of 12,018 healthy children aged 6–35 months and reported a 64% efficacy against moderate-to-severe influenza. In another large multinational RCT, Pepin et al. (37) reported QIV efficacy values of 51% (97%CI = 37%–62%) and 68% (97%CI = 47%–82%) against LCI caused by any influenza A or B strain and vaccine-similar strains, respectively. However, vaccine efficacy ranged from 41% to 60% during each of four influenza seasons, and the efficacy values for two QIV preparations were 46% (overall for the Southern Hemisphere 2014 and Northern Hemisphere 2014–2015 strains) and 58% (overall for the Southern 2015 and Northern 2015–2016 strains). A registry-based American study (124) in children aged 6–21 months carried out during the 2003–2004 season found that two doses of the vaccine were 69% and 87% effective against ILI and pneumonia/influenza office visits, respectively, but a single dose was not. Limited data on the efficacy estimates of IIVs exist in children <2 years. It is unclear whether the VE estimates are better for children <2 years old or those 2–5 years old (36) and impact indirect benefits for the communities by reducing transmission. Overall, data from test-negative design studies (29, 125–131) suggested moderate effectiveness of IIV against LCI in young children, but the confidence intervals are wide, suggesting imprecise estimates (**Tables 2**, **3**). A Canadian test-negative design study (152) found that the adjusted VE rates against LCI hospitalization were 60% (95%CI = 44%–72%) for fully vaccinated 6- to 59-month-old children and 39% (95%CI = 17%–56%) for partially vaccinated children. Another test-negative design study (153) found that the VE against LCI hospitalization was 46% (95%CI = 19%–64%) among children 6 months to <5 years old. These two studies suggested that annual influenza vaccination is 40%–60% effective against LCI hospitalization in young children; however, the estimates included LAIVs and were not restricted to IIVs. More recent studies (146–151) have rather found high VE rates against LCI hospitalization (up to 81%) in young children (**Table 2**).

**Table 2:** Vaccine efficacy and effectiveness of influenza vaccination in healthy children under 5 years of age.

Vaccine strains	Age (no. of participants)	Outcomes	Effect sizes (95%CI)	First author, year (region)
**RCTs**				
Seasonal TIV 1995–1996 NH, F	<6 years (*n* = 476)	Influenza RR ILI RR	0.61 (0.34–1.08) 0.39 (0.21–0.69)	Colombo, 2001; Clover, 1991; and Gruber, 1990 (132–135) (Italy and USA)
Seasonal TIV 1999–2000 N and 2000–2001 NH, H	<2 years (*n* = 786)	Influenza risk ratio	0.55 (0.18–1.69)	Hoberman, 2003 (135, 136) (Netherlands, two studies)
Seasonal TIV 1999–2000 and 2000–2001 NH subvirion, H	6–24 months (*n* = 786) Groups (first cohort: 411; second cohort: 375)	Efficacy against culture-confirmed influenza	First cohort: 66% (34–82) Second cohort: −7% (−247 to 67)	Hoberman, 2003 (136) (USA)
TIV 2003–2004, 2004–2005, and 2005–2006 N	18–72 months with H/O RTI (*n* = 579)	Efficacy against PCR-confirmed influenza	51% (3–75) despite substantial strain mismatch	Jansen, 2008 (137) (Netherlands)
Seasonal TIV 2007–2009 NH (MF59 emulsion adjuvant or subunit non-adjuvanted), F	6–72 months (*n* = 4,707)	Efficacy against all PCR-confirmed influenza strains for adjuvanted and non-adjuvanted TIV	86% (74–93) and 43% (15–61)	Vesikari, 2011 (59) (Finland and Germany)
Seasonal QIV 2010–2011, F	3–4 years (*n* = 80)	Efficacy against all PCR-confirmed influenza, any severity	35.3% (−1.3 to 58.6)	Jain, 2013 (138) (15 centers in Bangladesh, the Dominican Republic, Honduras, Lebanon, Panama, the Philippines, Thailand, and Turkey)
Seasonal TIV 2010–2014 SH, H	6–23 months (*n* = 4,081)	Efficacy against PCR-confirmed influenza	31% (18–42)	Rolfes, 2017 (139) (Bangladesh)
Five seasonal QIV formulations, F	6–35 months (*n* = 6,006 in QIV and *n* = 6,012 in control)	Efficacy against moderate-to-severe influenza and all influenza in total vaccinated cohorts	64% (53–73) 50% (42–57)	Claeys, 2018 (123) (13 countries in Europe, Central America, and Asia)
Seasonal 2008–2009 TIV NH	6 months to 10 years (*n* = 3,918 in TIV and *n* = 3,848 in control)	Adjusted VE in 6- to 35-month-olds and in 3- to 5-year-olds in preventing A/H3N2 and A/H1N1pdm09	20.6% (−16.3 to 45.8) and 57.7% (34.7–72.7); −30.8% (−128.3 to 25.0), and −56.2% (−238.2 to 27.8)	Diallo, 2019 (140) (Senegal)
Thimerosal-free, split-virion seasonal QIV NH (2014–2015 and 2015–2016) and SH (2014 and 2015) F; TIV, F	6–35 months (*n* = 4,980)	LCI caused by any influenza A or B strain and vaccine-similar strains	50.98% (37.36–61.86) and 68.40% (47.07–81.92)	Pepin, 2019 (37) (13 countries in Europe, Central America, and Asia)
Seasonal TIV 2009–2012 NH, H	6–35 months (*n* = 807)	Efficacy against PCR-confirmed influenza in 2011–2012	70.5% (24.2–88.5)	Sullender, 2019 (141) (India)
**Cohort studies**				
Seasonal 1998–1999 TIV NH subvirion	7–50 months (187 vaccinees and 187 controls)	Reduction in incidence of acute otitis media associated with influenza A	83% (58–93)	Heikkinen, 1991 (142) (Finland)
Five influenza seasons (from 2004–2005 to 2008–2009)	All aged 6–23 months (*n* = 919,021), full-term children (*n* = 847,294) and preterm children (*n* = 71,727)	Influenza-coded ambulatory visits VE in all children, full-term and preterm children (full and partial vaccination with an unvaccinated reference group)	19% (3–32) and 2% (−12 to 15) 18% (1–32) and 1% (−13 to 15) 28% (−29 to 60) and 4% (−50 to 38)	Shen, 2013 (30) (Canada)
Seasonal 2015–2018 TIV NH	2 years (*n* = 60,088 in 2015–2016, *n* = 60,860 in 2016–2017, *n* = 60,345 in 2017–2018)	VE against LCI caused by influenza strains (any, A and B) in 2015–2016, 2016–2017, and 2017–2018	77.2% (48.9–89.8), 90.3% (60.9–97.6), and 34.6% (−79.5 to 76.1); 24.5% (−29.8 to 56.1), 23.1% (−32.3 to 55.3), and NA; −20.1% (−61.5 to 10.7), −42.0% (−110.6 to 4.2), and −0.2% (−55.9 to 35.6)	Baum, 2020 (143) (Finland)
**Classic case–control studies**				
Seasonal TIV 1999–2006	6–59 months (15 fully vaccinated and 75 unvaccinated)	VE against LCI	86% (29–97)	Joshi, 2009 (36, 144) (USA)
Seasonal 2007–2008 TIV NH, F	9 months to 3 years (*n* = 340; 84 vaccinated and 256 unvaccinated)	VE against influenza A VE against influenza B VE any influenza	85% (37–96) 48% (−38 to 81) 72% (35–88)	Heinonen, 2011 (145) (Finland)
**Test-negative design^c^**				
2003–2005 IIV, F	6–59 months (*n* = 2,474)	VE against LCI in 2003–2004 (two and one dose) VE against LCI in 2004–2005 (two and one dose)	44% (−42 to 78) and 43% (−3 to 68) 57% (28–74) and 11% (−35 to 41)	Eisenberg, 2008 (125) (USA)
Adjuvanted pandemic 2009 H1N1 influenza vaccine, H	6–59 months (*n* = 53)	VE against LCI	100% (44.0–100)	Van Buynder, 2010 (130) (Canada)
Seasonal 2007–2008 TIV NH	6–59 months (*n* = 412)	VE against LCI	39% (2–62)	Belongia, 2011 (129) (USA)
Seasonal 2008 TIV SH, H (<3 years old) and F (3–5 years old)	6–59 months (*n* = 289)	Adjusted VE against LCI using all controls	58% (9–81)	Kelly, 2011 (128) (Australia)
Seasonal 2005–2007 TIV NH, F	6–59 months (*n* = 528)	VE against LCI for 6- to 59-month-olds Subgroups: 6–23 months and 24–59 months old	56% (25–74) 61% (16–82) and 56% (3–80)	Staat, 2011 (29) (Canada)
Seasonal 2008–2012 TIV	6–59 months (*n* = 1,514)	VE against LCI against influenza A Against influenza B	79.6% (41.6–92.9) 47.8% (−12.4 to 75.8)	Blyth, 2014 (126) (USA)
Seasonal 2009–2013 TIV^a^	≤2 years (*n* = 2,881) 3–5 years (*n* = 1,481)	VE against influenza A and B	34.1% (−27.3 to 65.9) 70.4% (36.3–86.3)	Cowling, 2014 (127) (Hong Kong)
Seasonal 2012-13 TIV^a^	6 months to 5 years (*n* = 334)	Adjusted VE against LCI hospitalizations	75% (−100 to 97)	Turner, 2014 (146) (New Zealand)
Seasonal 2013–2015 TIV NH^b^	6 months to 5 years (*n* = 665)	Adjusted VE against LCI hospitalizations	81.2% (−52.3 to 97.7)	Qin, 2016 (147) (China)
Seasonal 2015–2016 TIV NH^b^	<5 years (*n* = 832)	Adjusted VE against LCI hospitalizations	−63.7% (−423.6 to 48.9%)	Zhang, 2017 (148) (China)
Seasonal 2011–2015 TIV or QIV^a^	0.5–2 years (*n* = 12,516 fully *vs. n* = 11,949 partially vaccinated) 3–5 years (*n* = 7,295 fully *vs. n* = 6,467 partially vaccinated)	Adjusted VE against influenza A and B hospitalizations	74% (64–81%) *vs.* 18% (−20 to 43) 74% (68–80) *vs.* 47% (13–67)	Chua, 2019 (149) (Hong Kong)
Seasonal 2015–2018 TIV	6–24 months (*n* = 246 fully *vs. n* = 436 partially vaccinated) (*n* = 1,228 unvaccinated)	Adjusted VE against LCI hospitalizations for fully and partially vaccinated children	48.1% (8.3–72.6) and 9.3% (−27.1 to 40.9)	Segaloff, 2019 (150) (Israel)
Seasonal 2013–2017 TIV SH	6–24 months (*n* = 2,389)	Adjusted VE against LCI hospitalizations for fully and partially vaccinated children	43% (33–51) and 20% (−16 to 45)	Arriola, 2019 (151) (Argentina, Brazil, Chile, Colombia, and Paraguay)

Children 6 months to 5 years old require two doses as a prime–boost regime to ensure adequate seroprotection against influenza. Thereafter, only one annual dose is required. Fully vaccinated children received either two doses of IIV, or one dose in primed children and two doses in unprimed children, unless otherwise stated.

RCTs, randomized controlled trials; F, full dose (0.5 ml); H, half dose (0.25 ml); BIV, bivalent influenza vaccine; TIV, trivalent influenza vaccine; QIV, quadrivalent influenza vaccine; NH, WHO Northern Hemisphere recommended strains; RR, risk ratio; ILI, influenza-like illness; VE, vaccine effectiveness; LCI, laboratory-confirmed influenza; VA, vaccine arm; CA, control arm; RTI, respiratory tract infection.

a Estimates in 2009–2010 were also adjusted for receipt of the monovalent A(H1N1)pdm09 vaccine.

b Recruited patients who received at least one dose of influenza vaccine were identified as vaccinated.

c Test-negative design (117) used to evaluate influenza vaccine effectiveness.

**Table 3:** Weighing of the potential benefits against risks of harm from the inactivated influenza vaccine in pregnant women and young children.

Criteria	Pregnant women	Children aged 6 months to 5 years
Rationale for influenza vaccination	Risk of severe complications due to influenza infection	Risk of severe complications due to influenza infection, particularly in children less than 2 years of age High attack rates and the main source of influenza transmission High burden of influenza in low- and middle-income countries
Potential harm of inactivated influenza vaccine	Current literature does not suggest evidence of harm to the mother or fetus following inactivated influenza vaccine (IIV). Side effects, if any, are usually transient and minor.	Current literature does not suggest evidence of harm to the child following IIV. Side effects, if any, are usually transient and minor. In one study in China, IIV increased the risk of hospitalization in the 2015–2016 season, where a vaccine mismatch occurred (vaccine effectiveness: −63.7%; 95%CI = −423.6% to 48.9%, suggesting imprecise estimates)
Potential benefits of inactivated influenza vaccine	Moderate efficacy/effectiveness^a^ of IIV against laboratory-confirmed influenza (range = 50%–70%) and influenza-related hospitalization (range = 45–65%) Passive mother-to-child immunity protecting newborns from influenza and influenza-related hospitalization following maternal IIV Newborns born to vaccinated mothers are less likely to be premature, small for gestational age, and of low birth weight when compared to those of unvaccinated mothers.	Moderate to high efficacy/effectiveness^a^ of IIV against laboratory-confirmed influenza (range = 20%–90%) and influenza-related hospitalization (range = 43%–81% in those fully vaccinated) Preventing influenza transmission within the family and in the community
Acceptability to clinicians, pregnant women, and parents	Likely acceptable to pregnant women without contraindications, when shown appropriate risk-to-benefit ratio following influenza vaccination	Likely acceptable to children (parents/guardian) without contraindications, when shown appropriate risk-to-benefit ratio following influenza vaccination

a Determining exact IIV effectiveness estimates is challenging due to several factors, such as the IIV type (whole virus, virosome, split virus, or subunit) and manufacturing processes (eggs, cell culture, or recombinant DNA technologies) (31). Other confounding factors (31) are the vaccinee’s age, preexisting immunity, and comorbidities, as well as antigenic match/mismatches between the vaccine strains and circulating viruses and the use of adjuvants. Furthermore, the wide confidence intervals of the IIV effectiveness point estimates in most studies suggest imprecise knowledge.

Diallo et al. (140) found that TIV-mediated VE estimates were lower in children <3 years old compared to those in 3–10 years old in Senegal (21% *vs.* 60% against the predominant seasonal A/H3N2 strain). However, the authors did not demonstrate noteworthy indirect effects among the entire study villages, which may be attributed to the study design, low vaccine coverage, and the high participant contact rates between extended families (140). Moreover, the indirect effect was assessed by comparing those vaccinated with control villages, and the study was undertaken in a year of vaccine mismatch. On the other hand, a Canadian study (154) in vaccinated children (from 36 months to 15 years old) showed an indirect VE of 60% (95%CI = 8%–83%) among the unvaccinated in 49 Hutterite communities, similar to a VE of 59% (95%CI = 5%–82%). IIV-mediated beneficial effects were also seen for other clinical outcomes in children (101, 155), such as reduced medical visits and antibiotic use. The influenza VE against nonspecific clinical outcomes such as acute otitis media is expected to be lower than its effectiveness against LCI because acute otitis media is primarily caused by viral or bacterial co-infection (156) in children. A 2017 Cochrane systematic review (157) found that the influenza vaccine resulted in a small reduction (risk ratio = 0.84) in at least one episode of acute otitis media over at least 6 months in 3,134 children aged 6–36 months. In summary, administration of IIV in young children prevents influenza complications in the vaccinees and may reduce the community transmission of influenza (141, 154).

Additionally, one recent study found that TIV was associated with lower coronavirus disease 2019 (COVID-19)-related severity and mortality in children <5 years old in Brazil (158). However, this retrospective observational study had selection bias and constrained data as it did not include potentially confounding factors such as other vaccines; thus, further investigations are required to confirm the findings.

## Future Perspectives

A combined immunization strategy for pregnant women and young children who had IIVs may leverage both the direct protection of vaccinees and the indirect protection of non-vaccinees through vertically transmitted maternal antibodies and reduced person-to-person community transmission and mortality from influenza (4, 41, 159). However, conventional IIVs are grown in chicken eggs, with a long production time and impure contents. Previous literature suggested possible undesirable effects of egg-driven viral substitutions for optimal growth in eggs (31) and the induction of anti-egg antibodies in vaccinees (160, 161), which may have an impact on the vaccine efficacy, although no study has been done to evaluate this in pregnant women and young children. The long production times of IIVs could result in a vaccine antigen and circulating strain mismatch, affecting the VE estimates (162, 163), although studies have shown protection of IIVs in children despite vaccine strain mismatches (123). There is an urgent need for alternative methods for a more rapid production of vaccines. Cell-grown IIVs offer an alternative to egg-based vaccines. Cell-grown QIVs or TIVs have good immunogenicity profiles and may have a modest improvement over egg-based vaccines in children and adolescents (164, 165) while having similar safety profiles to those of egg-based IIVs in children (166, 167). One study found that the recombinant HA-based vaccine outperformed the virion-based IIV in both HA-specific cellular and serological responses in adults (168). However, to confirm these findings, larger RCTs are needed in pregnant women and younger children. In this review, the few relevant studies, along with major clinical, design, and statistical heterogeneities, precluded quantitative meta-analysis (4).

The ongoing COVID-19 pandemic has led to a paradigm shift in vaccinology, particularly with the use of new vaccine platforms that have not been previously licensed, especially messenger RNA (mRNA) vaccines. By 2019, 15 mRNA vaccine candidates, including three against influenza, were in clinical trials and none in phase III trials [review in (169)]. The COVID-19 pandemic has led to the most rapid vaccine development and approval of the mRNA COVID-19 vaccines to be used in humans. However, data on vaccine safety and efficacy in pregnant women are limited. Preliminary studies in pregnant and lactating women (170, 171) have suggested that the mRNA COVID-19 vaccines are safe, with immunogenicity and reactogenicity profiles similar to those observed in non-pregnant women. No data are yet available for young children since the mRNA COVID-19 vaccine clinical trials in children under 12 years of age are still ongoing. Nevertheless, the large safety database and good efficacy of the mRNA COVID-19 vaccines will accelerate the development of next-generation influenza vaccines, although it may well take over a decade before mRNA influenza vaccines will be licensed for pregnant women and young children. Therefore, the currently available IIVs should be continued to be recommended and used in pregnant women and young children until next-generation vaccines are available.

## Conclusions

Overall, studies demonstrate that pregnant women and young children are protected against influenza illness and hospitalization by IIVs. Current evidence suggests that the benefits of IIVs outweigh the potential risks and that IIVs should be offered to pregnant women and young children. Moderate efficacy/effectiveness estimates after influenza vaccination, with acceptable tolerability profiles, are observed in pregnant women and young children, and the immunogenicity profile of pregnant women is comparable with healthy adults. Vaccine efficacy/effectiveness estimates are similar after both the second and the third trimester vaccination in pregnant women, while these estimates are lower in young children. Limited data on vaccine efficacy/effectiveness estimates exist for the first trimester and for younger children, although vertical transmission of antibodies may protect newborns who are at high risk of influenza-related complications. Robust trials should evaluate newer generations of influenza vaccines, especially cell-grown vaccines, in pregnant women and young children as even a modest vaccine efficacy/effectiveness enhancement could translate into major clinical benefits.

## Author Contributions

AB, M-CT, and RC contributed to the conception and design of the study. AB wrote the first draft and amended the manuscript. M-CT, KM, and RC reviewed the manuscript and suggested amendments. All authors contributed to the article and approved the submitted version.

## Funding

This work was supported by a grant from the Research Council of Norway GLOBVAC program (284930) and the Ministry of Health and Care Services. AB and M-CT received research fellowships from the University of Bergen, Norway. This influenza center is funded by the Helse Vest (F-11628), the Trond Mohn Stiftelse (TMS2020TMT05), the European Union (EU IMI115672, FLUCOP, H2020 874866 INCENTIVE, and H2020 101037867 VACCELERATE), and Nanomedicines Flunanoair (ERA-NETet EuroNanoMed2 i JTC2016). The funders had no role in the preparation of the manuscript or in the decision to submit the manuscript for publication.

## Conflict of Interest

The authors declare that the research was conducted in the absence of any commercial or financial relationships that could be construed as a potential conflict of interest.

## Publisher’s Note

All claims expressed in this article are solely those of the authors and do not necessarily represent those of their affiliated organizations, or those of the publisher, the editors and the reviewers. Any product that may be evaluated in this article, or claim that may be made by its manufacturer, is not guaranteed or endorsed by the publisher.

## References

[1] Dolin R. Epidemiology of Influenza UpToDate. Waltham, MA: UpToDate (2020). Available at: https://www.uptodate.com/contents/epidemiology-of-influenza/print.

[2] The Centers for Disease Control and Prevention. Past Pandemics (2021). Available at: https://www.cdc.gov/flu/pandemic-resources/basics/past-pandemics.html.

[3] Krammer F Smith GJD Fouchier RAM Peiris M Kedzierska K Doherty PC. Influenza. Nat Rev Dis Primers (2018) 4(1):3. doi: 10.1038/s41572-018-0002-y 29955068 PMC7097467

[4] Fell DB Azziz-Baumgartner E Baker MG Batra M Beaute J Beutels P. Influenza Epidemiology and Immunization During Pregnancy: Final Report of a World Health Organization Working Group. Vaccine (2017) 35(43):5738–50. doi: 10.1016/j.vaccine.2017.08.037 PMC8274347 28867508

[5] Nair H Brooks WA Katz M Roca A Berkley JA Madhi SA. Global Burden of Respiratory Infections Due to Seasonal Influenza in Young Children: A Systematic Review and Meta-Analysis. Lancet (2011) 378(9807):1917–30. doi: 10.1016/S0140-6736(11)61051-9 22078723

[6] Doyle TJ Goodin K Hamilton JJ. Maternal and Neonatal Outcomes Among Pregnant Women With 2009 Pandemic Influenza A(H1N1) Illness in Florida, 2009-2010: A Population-Based Cohort Study. PloS One (2013) 8(10):e79040. doi: 10.1371/journal.pone.0079040 24205364 PMC3812024

[7] Yates L Pierce M Stephens S Mill AC Spark P Kurinczuk JJ. Influenza A/H1N1v in Pregnancy: An Investigation of the Characteristics and Management of Affected Women and the Relationship to Pregnancy Outcomes for Mother and Infant. Health Technol Assess (2010) 14(34):109–82. doi: 10.3310/hta14340-02 20630123

[8] Creanga AA Johnson TF Graitcer SB Hartman LK Al-Samarrai T Schwarz AG. Severity of 2009 Pandemic Influenza A (H1N1) Virus Infection in Pregnant Women. Obstet Gynecol (2010) 115(4):717–26. doi: 10.1097/AOG.0b013e3181d57947 20308830

[9] Jamieson DJ Honein MA Rasmussen SA Williams JL Swerdlow DL Biggerstaff MS. H1N1 2009 Influenza Virus Infection During Pregnancy in the USA. Lancet (2009) 374(9688):451–8. doi: 10.1016/S0140-6736(09)61304-0 19643469

[10] Knight M Pierce M Seppelt I Kurinczuk JJ Spark P Brocklehurst P. Critical Illness With AH1N1v Influenza in Pregnancy: A Comparison of Two Population-Based Cohorts. BJOG (2011) 118(2):232–9. doi: 10.1111/j.1471-0528.2010.02736.x 21040393

[11] World Health Organization. WHO. Influenza 2019. Available at: http://www.who.int/biologicals/vaccines/influenza/en/.

[12] Wang X Li Y O’Brien KL Madhi SA Widdowson MA Byass P. Global Burden of Respiratory Infections Associated With Seasonal Influenza in Children Under 5 Years in 2018: A Systematic Review and Modelling Study. Lancet Glob Health (2020) 8(4):e497–510. doi: 10.1016/S2214-109X(19)30545-5 PMC7083228 32087815

[13] Brooks WA Goswami D Rahman M Nahar K Fry AM Balish A. Influenza is a Major Contributor to Childhood Pneumonia in a Tropical Developing Country. Pediatr Infect Dis J (2010) 29(3):216–21. doi: 10.1097/INF.0b013e3181bc23fd 20190613

[14] Heikkinen T Silvennoinen H Peltola V Ziegler T Vainionpaa R Vuorinen T. Burden of Influenza in Children in the Community. J Infect Dis (2004) 190(8):1369–73. doi: 10.1086/424527 15378427

[15] Xu X Blanton L Elal AIA Alabi N Barnes J Biggerstaff M. Update: Influenza Activity in the United States During the 2018-19 Season and Composition of the 2019-20 Influenza Vaccine. MMWR Morb Mortal Wkly Rep (2019) 68(24):544–51. doi: 10.15585/mmwr.mm6824a3 PMC6586370 31220057

[16] Centers for Disease C, Prevention. Influenza Activity–United States, 2012-13 Season and Composition of the 2013-14 Influenza Vaccine. MMWR Morb Mortal Wkly Rep (2013) 62(23):473–9. PMC4604847 23760189

[17] Heikkinen T Tsolia M Finn A. Vaccination of Healthy Children Against Seasonal Influenza: A European Perspective. Pediatr Infect Dis J (2013) 32(8):881–8. doi: 10.1097/INF.0b013e3182918168 23856713

[18] Silvennoinen H Peltola V Vainionpää R Ruuskanen O Heikkinen T. Incidence of Influenza-Related Hospitalizations in Different Age Groups of Children in Finland: A 16-Year Study. Pediatr Infect Dis J (2011) 30(2):e24–8. doi: 10.1097/INF.0b013e3181fe37c8 21298851

[19] Nielsen J Krause TG Molbak K. Influenza-Associated Mortality Determined From All-Cause Mortality, Denmark 2010/11-2016/17: The FluMOMO Model. Influenza Other Respir Viruses (2018) 12(5):591–604. doi: 10.1111/irv.12564 29660769 PMC6086850

[20] Zhou H Thompson WW Viboud CG Ringholz CM Cheng PY Steiner C. Hospitalizations Associated With Influenza and Respiratory Syncytial Virus in the United States, 1993-2008. Clin Infect Dis (2012) 54(10):1427–36. doi: 10.1093/cid/cis211 PMC3334364 22495079

[21] Poehling KA Edwards KM Weinberg GA Szilagyi P Staat MA Iwane MK. The Underrecognized Burden of Influenza in Young Children. N Engl J Med (2006) 355(1):31–40. doi: 10.1056/NEJMoa054869 16822994

[22] Antonova EN Rycroft CE Ambrose CS Heikkinen T Principi N. Burden of Paediatric Influenza in Western Europe: A Systematic Review. BMC Public Health (2012) 12:968. doi: 10.1186/1471-2458-12-968 23146107 PMC3534559

[23] Krammer F. The Human Antibody Response to Influenza A Virus Infection and Vaccination. Nat Rev Immunol (2019) 19(6):383–97. doi: 10.1038/s41577-019-0143-6 30837674

[24] Frank AL Taber LH. Variation in Frequency of Natural Reinfection With Influenza A Viruses. J Med Virol (1983) 12(1):17–23. doi: 10.1002/jmv.1890120103 6619811

[25] Memoli MJ Han A Walters KA Czajkowski L Reed S Athota R. Influenza A Reinfection in Sequential Human Challenge: Implications for Protective Immunity and "Universal" Vaccine Development. Clin Infect Dis (2020) 70(5):748–53. doi: 10.1093/cid/ciz281 PMC7319262 30953061

[26] Gross PA Ennis FA Gaerlan PF Denson LJ Denning CR Schiffman D. A Controlled Double-Blind Comparison of Reactogenicity, Immunogenicity, and Protective Efficacy of Whole-Virus and Split-Product Influenza Vaccines in Children. J Infect Dis (1977) 136(5):623–32. doi: 10.1093/infdis/136.5.623 335000

[27] Young K GI Harrison R on behalf of the National Advisory Committee on Immunization (NACI). Summary of the NACI Seasonal Influenza Vaccine Statement for 2020–2021, (2020) 46:132–7. doi: 10.14745/ccdr.v46i05a06 PMC7279128 32558810

[28] Mak TK Mangtani P Leese J Watson JM Pfeifer D. Influenza Vaccination in Pregnancy: Current Evidence and Selected National Policies. Lancet Infect Dis (2008) 8(1):44–52. doi: 10.1016/S1473-3099(07)70311-0 18156088

[29] Staat MA Griffin MR Donauer S Edwards KM Szilagyi PG Weinberg GA. Vaccine Effectiveness for Laboratory-Confirmed Influenza in Children 6-59 Months of Age, 2005-2007. Vaccine (2011) 29(48):9005–11. doi: 10.1016/j.vaccine.2011.09.037 21945256

[30] Shen S Campitelli MA Calzavara A Guttmann A Kwong JC. Seasonal Influenza Vaccine Effectiveness in Pre- and Full-Term Children Aged 6-23 Months Over Multiple Seasons. Vaccine (2013) 31(29):2974–8. doi: 10.1016/j.vaccine.2013.05.011 23688524

[31] Paules CI Sullivan SG Subbarao K Fauci AS. Chasing Seasonal Influenza - The Need for a Universal Influenza Vaccine. N Engl J Med (2018) 378(1):7–9. doi: 10.1056/NEJMp1714916 29185857

[32] McHugh L Andrews RM Lambert SB Viney KA Wood N Perrett KP. Birth Outcomes for Australian Mother-Infant Pairs Who Received an Influenza Vaccine During Pregnancy, 2012-2014: The FluMum Study. Vaccine (2017) 35(10):1403–9. doi: 10.1016/j.vaccine.2017.01.075 28190746

[33] Tamma PD Ault KA del Rio C Steinhoff MC Halsey NA Omer SB. Safety of Influenza Vaccination During Pregnancy. Am J Obstet Gynecol (2009) 201(6):547–52. doi: 10.1016/j.ajog.2009.09.034 19850275

[34] Skowronski DM Hottes TS Chong M De Serres G Scheifele DW Ward BJ. Randomized Controlled Trial of Dose Response to Influenza Vaccine in Children Aged 6 to 23 Months. Pediatrics (2011) 128(2):e276–89. doi: 10.1542/peds.2010-2777 21768314

[35] Langley JM Vanderkooi OG Garfield HA Hebert J Chandrasekaran V Jain VK. Immunogenicity and Safety of 2 Dose Levels of a Thimerosal-Free Trivalent Seasonal Influenza Vaccine in Children Aged 6-35 Months: A Randomized, Controlled Trial. J Pediatr Infect Dis Soc (2012) 1(1):55–63. doi: 10.1093/jpids/pis012 PMC3656551 23687572

[36] Sullivan SG Price OH Regan AK. Burden, Effectiveness and Safety of Influenza Vaccines in Elderly, Paediatric and Pregnant Populations. Ther Adv Vaccines Immunother (2019) 7:2515135519826481. doi: 10.1177/2515135519826481 30793097 PMC6376509

[37] Pepin S Dupuy M Borja-Tabora CFC Montellano M Bravo L Santos J. Efficacy, Immunogenicity, and Safety of a Quadrivalent Inactivated Influenza Vaccine in Children Aged 6-35months: A Multi-Season Randomised Placebo-Controlled Trial in the Northern and Southern Hemispheres. Vaccine (2019) 37(13):1876–84. doi: 10.1016/j.vaccine.2018.11.074 30558818

[38] Del Giudice G Rappuoli R Didierlaurent AM. Correlates of Adjuvanticity: A Review on Adjuvants in Licensed Vaccines. Semin Immunol (2018) 39:14–21. doi: 10.1016/j.smim.2018.05.001 29801750

[39] Tunheim G Laake I Robertson AH Waalen K Hungnes O Naess LM. Antibody Levels in a Cohort of Pregnant Women After the 2009 Influenza A(H1N1) Pandemic: Waning and Association With Self-Reported Severity and Duration of Illness. Influenza Other Respir Viruses (2019) 13(2):191–200. doi: 10.1111/irv.12623 30536590 PMC6379636

[40] Hviid A Svanstrom H Molgaard-Nielsen D Lambach P. Association Between Pandemic Influenza A(H1N1) Vaccination in Pregnancy and Early Childhood Morbidity in Offspring. JAMA Pediatr (2017) 171(3):239–48. doi: 10.1001/jamapediatrics.2016.4023 27893898

[41] Zaman K Roy E Arifeen SE Rahman M Raqib R Wilson E. Effectiveness of Maternal Influenza Immunization in Mothers and Infants. N Engl J Med (2008) 359(15):1555–64. doi: 10.1056/NEJMoa0708630 18799552

[42] Munoz FM Greisinger AJ Wehmanen OA Mouzoon ME Hoyle JC Smith FA. Safety of Influenza Vaccination During Pregnancy. Am J Obstet Gynecol (2005) 192(4):1098–106. doi: 10.1016/j.ajog.2004.12.019 15846187

[43] Black SB Shinefield HR France EK Fireman BH Platt ST Shay D. Effectiveness of Influenza Vaccine During Pregnancy in Preventing Hospitalizations and Outpatient Visits for Respiratory Illness in Pregnant Women and Their Infants. Am J Perinatol (2004) 21(6):333–9. doi: 10.1055/s-2004-831888 15311370

[44] Yeager DP Toy EC Baker B. Influenza Vaccination in Pregnancy. Am J Perinatol (1999) 16(6):283–6. doi: 10.1055/s-2007-993873 10586981

[45] Englund JA Mbawuike IN Hammill H Holleman MC Baxter BD Glezen WP. Maternal Immunization With Influenza or Tetanus Toxoid Vaccine for Passive Antibody Protection in Young Infants. J Infect Dis (1993) 168(3):647–56. doi: 10.1093/infdis/168.3.647 8354906

[46] Deinard AS Ogburn P. A/NJ/8/76 Influenza Vaccination Program: Effects on Maternal Health and Pregnancy Outcome. Am J Obstet Gynecol (1981) 140(3):240–5. doi: 10.1016/0002-9378(81)90267-2 7246624

[47] Sumaya CV Gibbs RS. Immunization of Pregnant Women With Influenza A/New Jersey/76 Virus Vaccine: Reactogenicity and Immunogenicity in Mother and Infant. J Infect Dis (1979) 140(2):141–6. doi: 10.1093/infdis/140.2.141 479636

[48] Donahue JG Kieke BA King JP Mascola MA Shimabukuro TT DeStefano F. Inactivated Influenza Vaccine and Spontaneous Abortion in the Vaccine Safety Datalink in 2012-13, 2013-14, and 2014-15. Vaccine (2019) 37(44):6673–81. doi: 10.1016/j.vaccine.2019.09.035 PMC6906603 31540812

[49] Vesikari T Virta M Heinonen S Eymin C Lavis N Chabanon AL. Immunogenicity and Safety of a Quadrivalent Inactivated Influenza Vaccine in Pregnant Women: A Randomized, Observer-Blind Trial. Hum Vaccin Immunother (2020) 16(3):623–9. doi: 10.1080/21645515.2019.1667202 PMC7227680 31526225

[50] Walsh LK Donelle J Dodds L Hawken S Wilson K Benchimol EI. Health Outcomes of Young Children Born to Mothers Who Received 2009 Pandemic H1N1 Influenza Vaccination During Pregnancy: Retrospective Cohort Study. BMJ (2019) 366:l4151. doi: 10.1136/bmj.l4151 31292120 PMC6614795

[51] Sukumaran L McCarthy NL Kharbanda EO Vazquez-Benitez G Lipkind HS Jackson L. Infant Hospitalizations and Mortality After Maternal Vaccination. Pediatrics (2018) 141(3):e20173310. doi: 10.1542/peds.2017-3310 29463582 PMC6586222

[52] Pillsbury A Cashman P Leeb A Regan A Westphal D Snelling T. Real-Time Safety Surveillance of Seasonal Influenza Vaccines in Children, Australia, 2015. Euro Surveill (2015) 20(43):30050. doi: 10.2807/1560-7917.ES.2015.20.43.30050 26536867

[53] Lina B Fletcher MA Valette M Saliou P Aymard M. A TritonX-100-Split Virion Influenza Vaccine Is Safe and Fulfills the Committee for Proprietary Medicinal Products (CPMP) Recommendations for the European Community for Immunogenicity, in Children, Adults and the Elderly. Biologicals (2000) 28(2):95–103. doi: 10.1006/biol.2000.0245 10885616

[54] Englund JA Walter EB Fairchok MP Monto AS Neuzil KM. A Comparison of 2 Influenza Vaccine Schedules in 6- to 23-Month-Old Children. Pediatrics (2005) 115(4):1039–47. doi: 10.1542/peds.2004-2373 15805382

[55] King JC Cox MM Reisinger K Hedrick J Graham I Patriarca P. Evaluation of the Safety, Reactogenicity and Immunogenicity of FluBlok Trivalent Recombinant Baculovirus-Expressed Hemagglutinin Influenza Vaccine Administered Intramuscularly to Healthy Children Aged 6-59 Months. Vaccine (2009) 27(47):6589–94. doi: 10.1016/j.vaccine.2009.08.032 19716456

[56] Walter EB Neuzil KM Zhu Y Fairchok MP Gagliano ME Monto AS. Influenza Vaccine Immunogenicity in 6- to 23-Month-Old Children: Are Identical Antigens Necessary for Priming? Pediatrics (2006) 118(3):e570–8. doi: 10.1542/peds.2006-0198 16950948

[57] Englund JA Walter E Black S Blatter M Nyberg J Ruben FL. Safety and Immunogenicity of Trivalent Inactivated Influenza Vaccine in Infants: A Randomized Double-Blind Placebo-Controlled Study. Pediatr Infect Dis J (2010) 29(2):105–10. doi: 10.1097/INF.0b013e3181b84c34 19934787

[58] Vesikari T Pellegrini M Karvonen A Groth N Borkowski A O’Hagan DT. Enhanced Immunogenicity of Seasonal Influenza Vaccines in Young Children Using MF59 Adjuvant. Pediatr Infect Dis J (2009) 28(7):563–71. doi: 10.1097/INF.0b013e31819d6394 19561422

[59] Vesikari T Knuf M Wutzler P Karvonen A Kieninger-Baum D Schmitt HJ. Oil-in-Water Emulsion Adjuvant With Influenza Vaccine in Young Children. N Engl J Med (2011) 365(15):1406–16. doi: 10.1056/NEJMoa1010331 21995388

[60] Esposito S Marchisio P Ansaldi F Bianchini S Pacei M Baggi E. A Randomized Clinical Trial Assessing Immunogenicity and Safety of a Double Dose of Virosomal-Adjuvanted Influenza Vaccine Administered to Unprimed Children Aged 6-35 Months. Vaccine (2010) 28(38):6137–44. doi: 10.1016/j.vaccine.2010.07.041 20670909

[61] Li-Kim-Moy J Yin JK Rashid H Khandaker G King C Wood N. Systematic Review of Fever, Febrile Convulsions and Serious Adverse Events Following Administration of Inactivated Trivalent Influenza Vaccines in Children. Euro Surveill (2015) 20(24). 10.2807/1560-7917.es2015.20.24.21159 26111238

[62] Watanabe T. Henoch-Schönlein Purpura Following Influenza Vaccinations During the Pandemic of Influenza A (H1n1). Pediatr Nephrol (2011) 26(5):795–8. doi: 10.1007/s00467-010-1722-8 21120537

[63] Goodman MJ Nordin JD Harper P Defor T Zhou X. The Safety of Trivalent Influenza Vaccine Among Healthy Children 6 to 24 Months of Age. Pediatrics (2006) 117(5):e821–6. doi: 10.1542/peds.2005-2234 16651286

[64] Halsey NA Talaat KR Greenbaum A Mensah E Dudley MZ Proveaux T. The Safety of Influenza Vaccines in Children: An Institute for Vaccine Safety White Paper. Vaccine (2015) 33(Suppl 5):F1–F67. doi: 10.1016/j.vaccine.2015.10.080 26822822

[65] Stassijns J Bollaerts K Baay M Verstraeten T. A Systematic Review and Meta-Analysis on the Safety of Newly Adjuvanted Vaccines Among Children. Vaccine (2016) 34(6):714–22. doi: 10.1016/j.vaccine.2015.12.024 26740250

[66] Brady RC Hu W Houchin VG Eder FS Jackson KC Hartel GF. Randomized Trial to Compare the Safety and Immunogenicity of CSL Limited’s 2009 Trivalent Inactivated Influenza Vaccine to an Established Vaccine in United States Children. Vaccine (2014) 32(52):7141–7. doi: 10.1016/j.vaccine.2014.10.024 25454878

[67] Armstrong PK Dowse GK Effler PV Carcione D Blyth CC Richmond PC. Epidemiological Study of Severe Febrile Reactions in Young Children in Western Australia Caused by a 2010 Trivalent Inactivated Influenza Vaccine. BMJ Open (2011) 1(1):e000016. doi: 10.1136/bmjopen-2010-000016 PMC3191393 22021725

[68] Tse A Tseng HF Greene SK Vellozzi C Lee GM Group VRCAIW. Signal Identification and Evaluation for Risk of Febrile Seizures in Children Following Trivalent Inactivated Influenza Vaccine in the Vaccine Safety Datalink Project, 2010-2011. Vaccine (2012) 30(11):2024–31. doi: 10.1016/j.vaccine.2012.01.027 22361304

[69] Pillsbury AJ Glover C Jacoby P Quinn HE Fathima P Cashman P. Active Surveillance of 2017 Seasonal Influenza Vaccine Safety: An Observational Cohort Study of Individuals Aged 6 Months and Older in Australia. BMJ Open (2018) 8(10):e023263. doi: 10.1136/bmjopen-2018-023263 PMC6196842 30341132

[70] Mohammed H Roberts CT Grzeskowiak LE Giles LC Dekker GA Marshall HS. Safety and Protective Effects of Maternal Influenza Vaccination on Pregnancy and Birth Outcomes: A Prospective Cohort Study. EClinicalMedicine (2020) 26:100522. doi: 10.1016/j.eclinm.2020.100522 32964200 PMC7490992

[71] Vesikari T Groth N Karvonen A Borkowski A Pellegrini M. MF59-Adjuvanted Influenza Vaccine (FLUAD) in Children: Safety and Immunogenicity Following a Second Year Seasonal Vaccination. Vaccine (2009) 27(45):6291–5. doi: 10.1016/j.vaccine.2009.02.004 19840662

[72] Wijnans L Lecomte C de Vries C Weibel D Sammon C Hviid A. The Incidence of Narcolepsy in Europe: Before, During, and After the Influenza A(H1N1)pdm09 Pandemic and Vaccination Campaigns. Vaccine (2013) 31(8):1246–54. doi: 10.1016/j.vaccine.2012.12.015 23246544

[73] Folkenberg M Callréus T Svanström H Valentiner-Branth P Hviid A. Spontaneous Reporting of Adverse Events Following Immunisation Against Pandemic Influenza in Denmark November 2009-March 2010. Vaccine (2011) 29(6):1180–4. doi: 10.1016/j.vaccine.2010.12.008 21172382

[74] Tokars JI Lewis P DeStefano F Wise M Viray M Morgan O. The Risk of Guillain-Barré Syndrome Associated With Influenza A (H1N1) 2009 Monovalent Vaccine and 2009-2010 Seasonal Influenza Vaccines: Results From Self-Controlled Analyses. Pharmacoepidemiol Drug Saf (2012) 21(5):546–52. doi: 10.1002/pds.3220 22407672

[75] Wise ME Viray M Sejvar JJ Lewis P Baughman AL Connor W. Guillain-Barre Syndrome During the 2009-2010 H1N1 Influenza Vaccination Campaign: Population-Based Surveillance Among 45 Million Americans. Am J Epidemiol (2012) 175(11):1110–9. doi: 10.1093/aje/kws196 PMC3888111 22582209

[76] Greene SK Rett M Weintraub ES Li L Yin R Amato AA. Risk of Confirmed Guillain-Barre Syndrome Following Receipt of Monovalent Inactivated Influenza A (H1N1) and Seasonal Influenza Vaccines in the Vaccine Safety Datalink Project, 2009-2010. Am J Epidemiol (2012) 175(11):1100–9. doi: 10.1093/aje/kws195 PMC6272801 22582210

[77] Petráš M Králová Lesná I Dáňová J Čelko AM. Is an Increased Risk of Developing Guillain–Barré Syndrome Associated With Seasonal Influenza Vaccination? A Systematic Review and Meta-Analysis. Vaccines (2020) 8(2):150. doi: 10.3390/vaccines8020150 PMC7349742 32230964

[78] Cox RJ. Correlates of Protection to Influenza Virus, Where do We Go From Here? Hum Vaccin Immunother (2013) 9(2):405–8. doi: 10.4161/hv.22908 PMC3859764 23291930

[79] Black S Nicolay U Vesikari T Knuf M Del Giudice G Della Cioppa G. Hemagglutination Inhibition Antibody Titers as a Correlate of Protection for Inactivated Influenza Vaccines in Children. Pediatr Infect Dis J (2011) 30(12):1081–5. doi: 10.1097/INF.0b013e3182367662 21983214

[80] Gianchecchi E Torelli A Montomoli E. The Use of Cell-Mediated Immunity for the Evaluation of Influenza Vaccines: An Upcoming Necessity. Hum Vaccin Immunother (2019) 15(5):1021–30. doi: 10.1080/21645515.2019.1565269 PMC6605831 30614754

[81] The European Agency for the Evaluation of Medicinal Products. Committee for Proprietary Medicinal Products. Note for Guidance on Harmonization of Requirements for Influenza Vaccines (1997). Available at: http://www.emea.europa.eu/docs/en_GB/document_library/Scientific_guideline/2009/09/WC500003945.pdf.

[82] Wang B Russell ML Brewer A Newton J Singh P Ward BJ. Single Radial Haemolysis Compared to Haemagglutinin Inhibition and Microneutralization as a Correlate of Protection Against Influenza A H3N2 in Children and Adolescents. Influenza Other Respir Viruses (2017) 11(3):283–8. doi: 10.1111/irv.12450 PMC5410716 28218983

[83] Wijnans L Voordouw B. A Review of the Changes to the Licensing of Influenza Vaccines in Europe. Influenza Other Respir Viruses (2016) 10(1):2–8. doi: 10.1111/irv.12351 26439108 PMC4687503

[84] Kay AW Blish CA. Immunogenicity and Clinical Efficacy of Influenza Vaccination in Pregnancy. Front Immunol (2015) 6:289. doi: 10.3389/fimmu.2015.00289 26089824 PMC4455389

[85] Madhi SA Cutland CL Kuwanda L Weinberg A Hugo A Jones S. Influenza Vaccination of Pregnant Women and Protection of Their Infants. N Engl J Med (2014) 371(10):918–31. doi: 10.1056/NEJMoa1401480 25184864

[86] Nunes MC Cutland CL Jones S Hugo A Madimabe R Simoes EA. Duration of Infant Protection Against Influenza Illness Conferred by Maternal Immunization: Secondary Analysis of a Randomized Clinical Trial. JAMA Pediatr (2016) 170(9):840–7. doi: 10.1001/jamapediatrics.2016.0921 27380464

[87] Sperling RS Engel SM Wallenstein S Kraus TA Garrido J Singh T. Immunogenicity of Trivalent Inactivated Influenza Vaccination Received During Pregnancy or Postpartum. Obstet Gynecol (2012) 119(3):631–9. doi: 10.1097/AOG.0b013e318244ed20 PMC3327739 22353963

[88] Steinhoff MC Omer SB Roy E Arifeen SE Raqib R Altaye M. Influenza Immunization in Pregnancy–Antibody Responses in Mothers and Infants. N Engl J Med (2010) 362(17):1644–6. doi: 10.1056/NEJMc0912599 20427817

[89] Christian LM Porter K Karlsson E Schultz-Cherry S Iams JD. Serum Proinflammatory Cytokine Responses to Influenza Virus Vaccine Among Women During Pregnancy Versus Non-Pregnancy. Am J Reprod Immunol (2013) 70(1):45–53. doi: 10.1111/aji.12117 23551710 PMC3878156

[90] Nunes MC Weinberg A Cutland CL Jones S Wang D Dighero-Kemp B. Neutralization and Hemagglutination-Inhibition Antibodies Following Influenza Vaccination of HIV-Infected and HIV-Uninfected Pregnant Women. PloS One (2018) 13(12):e0210124. doi: 10.1371/journal.pone.0210124 30596775 PMC6312282

[91] Eick AA Uyeki TM Klimov A Hall H Reid R Santosham M. Maternal Influenza Vaccination and Effect on Influenza Virus Infection in Young Infants. Arch Pediatr Adolesc Med (2011) 165(2):104–11. doi: 10.1001/archpediatrics.2010.192 20921345

[92] Abbas AK Lichtman AH Pillai S Baker DL Baker A. Cellular and Molecular Immunology. Ninth edition. ed. Philadelphia, PA: Elsevier (2018). p. 565.

[93] Nunes MC Madhi SA. Prevention of Influenza-Related Illness in Young Infants by Maternal Vaccination During Pregnancy. F1000Res (2018) 7:122. doi: 10.12688/f1000research.12473.1 29445450 PMC5791002

[94] Malek A Sager R Kuhn P Nicolaides KH Schneider H. Evolution of Maternofetal Transport of Immunoglobulins During Human Pregnancy. Am J Reprod Immunol (1996) 36(5):248–55. doi: 10.1111/j.1600-0897.1996.tb00172.x 8955500

[95] Cuningham W Geard N Fielding JE Braat S Madhi SA Nunes MC. Optimal Timing of Influenza Vaccine During Pregnancy: A Systematic Review and Meta-Analysis. Influenza Other Respir Viruses (2019) 13(5):438–52. doi: 10.1111/irv.12649 PMC6692549 31165580

[96] Kittikraisak W Phadungkiatwatana P Ditsungnoen D Kaoiean S Macareo L Rungrojcharoenkit K. Comparison of Influenza Antibody Titers Among Women Who Were Vaccinated in the 2. Vaccine (2021) 39(1):18–25. doi: 10.1016/j.vaccine.2020.11.032 33243634

[97] Halasa NB Gerber MA Berry AA Anderson EL Winokur P Keyserling H. Safety and Immunogenicity of Full-Dose Trivalent Inactivated Influenza Vaccine (TIV) Compared With Half-Dose TIV Administered to Children 6 Through 35 Months of Age. J Pediatr Infect Dis Soc (2015) 4(3):214–24. doi: 10.1093/jpids/piu061 PMC4554205 26334249

[98] Walter EB Rajagopal S Zhu Y Neuzil KM Fairchok MP Englund JA. Trivalent Inactivated Influenza Vaccine (TIV) Immunogenicity in Children 6 Through 23 Months of Age: Do Children of All Ages Respond Equally? Vaccine (2010) 28(27):4376–83. doi: 10.1016/j.vaccine.2010.04.058 20447477

[99] Walter EB Englund JA Blatter M Nyberg J Ruben FL Decker MD. Trivalent Inactivated Influenza Virus Vaccine Given to Two-Month-Old Children: An Off-Season Pilot Study. Pediatr Infect Dis J (2009) 28(12):1099–104. doi: 10.1097/INF.0b013e3181b0c0ca 19935270

[100] Pepin S Szymanski H Rochin Kobashi IA Villagomez Martinez S Gonzalez Zamora JF Brzostek J. Safety and Immunogenicity of an Intramuscular Quadrivalent Influenza Vaccine in Children 3 to 8 Y of Age: A Phase III Randomized Controlled Study. Hum Vaccin Immunother (2016) 12(12):3072–8. doi: 10.1080/21645515.2016.1212143 PMC5215516 27565435

[101] Montomoli E Torelli A Manini I Gianchecchi E. Immunogenicity and Safety of the New Inactivated Quadrivalent Influenza Vaccine Vaxigrip Tetra: Preliminary Results in Children >/=6 Months and Older Adults. Vaccines (Basel) (2018) 6(1):14. doi: 10.3390/vaccines6010014 PMC5874655 29518013

[102] Wang W Chen Q Ford-Siltz LA Katzelnick LC Parra GI Song HS. Neutralizing Antibody Responses to Homologous and Heterologous H1 and H3 Influenza A Strains After Vaccination With Inactivated Trivalent Influenza Vaccine Vary With Age and Prior-Year Vaccination. Clin Infect Dis (2019) 68(12):2067–78. doi: 10.1093/cid/ciy818 30256912

[103] Omer SB. Maternal Immunization. N Engl J Med (2017) 376(13):1256–67. doi: 10.1056/NEJMra1509044 28355514

[104] Neuzil KM Mellen BG Wright PF Mitchel EF Jr Griffin MR. The Effect of Influenza on Hospitalizations, Outpatient Visits, and Courses of Antibiotics in Children. N Engl J Med (2000) 342(4):225–31. doi: 10.1056/NEJM200001273420401 10648763

[105] Thompson MG Li DK Shifflett P Sokolow LZ Ferber JR Kurosky S. Effectiveness of Seasonal Trivalent Influenza Vaccine for Preventing Influenza Virus Illness Among Pregnant Women: A Population-Based Case-Control Study During the 2010-2011 and 2011-2012 Influenza Seasons. Clin Infect Dis (2014) 58(4):449–57. doi: 10.1093/cid/cit750 24280090

[106] Regan AK Klerk N Moore HC Omer SB Shellam G Effler PV. Effectiveness of Seasonal Trivalent Influenza Vaccination Against Hospital-Attended Acute Respiratory Infections in Pregnant Women: A Retrospective Cohort Study. Vaccine (2016) 34(32):3649–56. doi: 10.1016/j.vaccine.2016.05.032 27216758

[107] Jarvis JR Dorey RB Warricker FDM Alwan NA Jones CE. The Effectiveness of Influenza Vaccination in Pregnancy in Relation to Child Health Outcomes: Systematic Review and Meta-Analysis. Vaccine (2020) 38(7):1601–13. doi: 10.1016/j.vaccine.2019.12.056 31932138

[108] Tapia MD Sow SO Tamboura B Tégueté I Pasetti MF Kodio M. Maternal Immunisation With Trivalent Inactivated Influenza Vaccine for Prevention of Influenza in Infants in Mali: A Prospective, Active-Controlled, Observer-Blind, Randomised Phase 4 Trial. Lancet Infect Dis (2016) 16(9):1026–35. doi: 10.1016/S1473-3099(16)30054-8 PMC4985566 27261067

[109] Steinhoff MC Katz J Englund JA Khatry SK Shrestha L Kuypers J. Year-Round Influenza Immunisation During Pregnancy in Nepal: A Phase 4, Randomised, Placebo-Controlled Trial. Lancet Infect Dis (2017) 17(9):981–9. doi: 10.1016/S1473-3099(17)30252-9 PMC5573632 28522338

[110] Katz J Englund JA Steinhoff MC Khatry SK Shrestha L Kuypers J. Impact of Timing of Influenza Vaccination in Pregnancy on Transplacental Antibody Transfer, Influenza Incidence, and Birth Outcomes: A Randomized Trial in Rural Nepal. Clin Infect Dis (2018) 67(3):334–40. doi: 10.1093/cid/ciy090 PMC6051462 29452372

[111] Poehling KA Szilagyi PG Staat MA Snively BM Payne DC Bridges CB. Impact of Maternal Immunization on Influenza Hospitalizations in Infants. Am J Obstet Gynecol (2011) 204(6 Suppl 1):S141–8. doi: 10.1016/j.ajog.2011.02.042 PMC3111909 21492825

[112] Sugimura T Nagai T Kobayashi H Ozaki Y Yamakawa R Hirata R. Effectiveness of Maternal Influenza Immunization in Young Infants in Japan. Pediatr Int (2016) 58(8):709–13. doi: 10.1111/ped.12888 26670462

[113] Dabrera G Zhao H Andrews N Begum F Green H Ellis J. Effectiveness of Seasonal Influenza Vaccination During Pregnancy in Preventing Influenza Infection in Infants, England, 2013/14. Euro Surveill (2014) 19(45):20959. doi: 10.2807/1560-7917.ES2014.19.45.20959 25411687

[114] Walker JL Zhao H Dabrera G Andrews N Thomas SL Tsang C. Assessment of Effectiveness of Seasonal Influenza Vaccination During Pregnancy in Preventing Influenza Infection in Infants in England, 2013-2014 and 2014-2015. J Infect Dis (2020) 221(1):16–20. 31711165 10.1093/infdis/jiz310

[115] Benowitz I Esposito DB Gracey KD Shapiro ED Vazquez M. Influenza Vaccine Given to Pregnant Women Reduces Hospitalization Due to Influenza in Their Infants. Clin Infect Dis (2010) 51(12):1355–61. doi: 10.1086/657309 PMC3106242 21058908

[116] Molgaard-Nielsen D Fischer TK Krause TG Hviid A. Effectiveness of Maternal Immunization With Trivalent Inactivated Influenza Vaccine in Pregnant Women and Their Infants. J Intern Med (2019) 286(4):469–80. doi: 10.1111/joim.12947 31259452

[117] Sullivan SG Feng S Cowling BJ. Potential of the Test-Negative Design for Measuring Influenza Vaccine Effectiveness: A Systematic Review. Expert Rev Vaccines (2014) 13(12):1571–91. doi: 10.1586/14760584.2014.966695 PMC4277796 25348015

[118] Shakib JH Korgenski K Presson AP Sheng X Varner MW Pavia AT. Influenza in Infants Born to Women Vaccinated During Pregnancy. Pediatrics (2016) 137(6):e20152360. doi: 10.1542/peds.2015-2360 27244843 PMC4894254

[119] Haberg SE Trogstad L Gunnes N Wilcox AJ Gjessing HK Samuelsen SO. Risk of Fetal Death After Pandemic Influenza Virus Infection or Vaccination. N Engl J Med (2013) 368(4):333–40. doi: 10.1056/NEJMoa1207210 PMC3602844 23323868

[120] Yamada T Yamada T Morikawa M Cho K Endo T Sato SS. Pandemic (H1N1) 2009 in Pregnant Japanese Women in Hokkaido. J Obstet Gynaecol Res (2012) 38(1):130–6. doi: 10.1111/j.1447-0756.2011.01644.x 21955086

[121] Jackson LA Patel SM Swamy GK Frey SE Creech CB Munoz FM. Immunogenicity of an Inactivated Monovalent 2009 H1N1 Influenza Vaccine in Pregnant Women. J Infect Dis (2011) 204(6):854–63. doi: 10.1093/infdis/jir440 PMC3156926 21849282

[122] Nunes MC Aqil AR Omer SB Madhi SA. The Effects of Influenza Vaccination During Pregnancy on Birth Outcomes: A Systematic Review and Meta-Analysis. Am J Perinatol (2016) 33(11):1104–14. doi: 10.1055/s-0036-1586101 27603545

[123] Claeys C Zaman K Dbaibo G Li P Izu A Kosalaraksa P. Prevention of Vaccine-Matched and Mismatched Influenza in Children Aged 6–35 Months: A Multinational Randomised Trial Across Five Influenza Seasons. Lancet Child Adolesc Health (2018) 2(5):338–49. doi: 10.1016/S2352-4642(18)30062-2 30169267

[124] Allison MA Daley MF Crane LA Barrow J Beaty BL Allred N. Influenza Vaccine Effectiveness in Healthy 6- to 21-Month-Old Children During the 2003-2004 Season. J Pediatr (2006) 149(6):755–62. doi: 10.1016/j.jpeds.2006.06.036 17137887

[125] Eisenberg KW Szilagyi PG Fairbrother G Griffin MR Staat M Shone LP. Vaccine Effectiveness Against Laboratory-Confirmed Influenza in Children 6 to 59 Months of Age During the 2003-2004 and 2004-2005 Influenza Seasons. Pediatrics (2008) 122(5):911–9. doi: 10.1542/peds.2007-3304 PMC3695734 18977968

[126] Blyth CC Jacoby P Effler PV Kelly H Smith DW Robins C. Effectiveness of Trivalent Flu Vaccine in Healthy Young Children. Pediatrics (2014) 133(5):e1218–25. doi: 10.1542/peds.2013-3707 24753525

[127] Cowling BJ Chan KH Feng S Chan EL Lo JY Peiris JS. The Effectiveness of Influenza Vaccination in Preventing Hospitalizations in Children in Hong Kong, 2009-2013. Vaccine (2014) 32(41):5278–84. doi: 10.1016/j.vaccine.2014.07.084 PMC4165553 25092636

[128] Kelly H Jacoby P Dixon GA Carcione D Williams S Moore HC. Vaccine Effectiveness Against Laboratory-Confirmed Influenza in Healthy Young Children: A Case-Control Study. Pediatr Infect Dis J (2011) 30(2):107–11. doi: 10.1097/INF.0b013e318201811c 21079528

[129] Belongia EA Kieke BA Donahue JG Coleman LA Irving SA Meece JK. Influenza Vaccine Effectiveness in Wisconsin During the 2007-08 Season: Comparison of Interim and Final Results. Vaccine (2011) 29(38):6558–63. doi: 10.1016/j.vaccine.2011.07.002 21767593

[130] Van Buynder PG Dhaliwal JK Van Buynder JL Couturier C Minville-Leblanc M Garceau R. Protective Effect of Single-Dose Adjuvanted Pandemic Influenza Vaccine in Children. Influenza Other Respir Viruses (2010) 4(4):171–8. doi: 10.1111/j.1750-2659.2010.00146.x PMC5964543 20629771

[131] Blyth CC Cheng AC Crawford NW Clark JE Buttery JP Marshall HS. The Impact of New Universal Child Influenza Programs in Australia: Vaccine Coverage, Effectiveness and Disease Epidemiology in Hospitalised Children in 2018. Vaccine (2020) 38(13):2779–87. doi: 10.1016/j.vaccine.2020.02.031 32107062

[132] Clover RD Crawford S Glezen WP Taber LH Matson CC Couch RB. Comparison of Heterotypic Protection Against Influenza A/Taiwan/86 (H1N1) by Attenuated and Inactivated Vaccines to A/Chile/83-Like Viruses. J Infect Dis (1991) 163(2):300–4. doi: 10.1093/infdis/163.2.300 1988512

[133] Colombo C Argiolas L La Vecchia C Negri E Meloni G Meloni T. Influenza Vaccine in Healthy Preschool Children. Rev Epidemiol Sante Publique (2001) 49(2):157–62. 11319482

[134] Gruber WC Taber LH Glezen WP Clover RD Abell TD Demmler RW. Live Attenuated and Inactivated Influenza Vaccine in School-Age Children. Am J Dis Child (1990) 144(5):595–600. doi: 10.1001/archpedi.1990.02150290089035 2330929

[135] Jefferson T Rivetti A Di Pietrantonj C Demicheli V. Vaccines for Preventing Influenza in Healthy Children. Cochrane Database Syst Rev (2018) 2:CD004879. doi: 10.1002/14651858.CD004879.pub5 29388195 PMC6491174

[136] Hoberman A Greenberg DP Paradise JL Rockette HE Lave JR Kearney DH. Effectiveness of Inactivated Influenza Vaccine in Preventing Acute Otitis Media in Young Children - A Randomized Controlled Trial. JAMA-J Am Med Assoc (2003) 290(12):1608–16. doi: 10.1001/jama.290.12.1608 14506120

[137] Jansen AG Sanders EA Hoes AW van Loon AM Hak E. Effects of Influenza Plus Pneumococcal Conjugate Vaccination Versus Influenza Vaccination Alone in Preventing Respiratory Tract Infections in Children: A Randomized, Double-Blind, Placebo-Controlled Trial. J Pediatr (2008) 153(6):764–70. doi: 10.1016/j.jpeds.2008.05.060 PMC7172528 18621393

[138] Jain VK Rivera L Zaman K Espos RA Sirivichayakul C Quiambao BP. Vaccine for Prevention of Mild and Moderate-to-Severe Influenza in Children. N Engl J Med (2013) 369(26):2481–91. doi: 10.1056/NEJMoa1215817 24328444

[139] Rolfes MA Goswami D Sharmeen AT Yeasmin S Parvin N Nahar K. Efficacy of Trivalent Influenza Vaccine Against Laboratory-Confirmed Influenza Among Young Children in a Randomized Trial in Bangladesh. Vaccine (2017) 35(50):6967–76. doi: 10.1016/j.vaccine.2017.10.074 PMC5723570 29100706

[140] Diallo A Diop OM Diop D Niang MN Sugimoto JD Ortiz JR. Effectiveness of Seasonal Influenza Vaccination in Children in Senegal During a Year of Vaccine Mismatch: A Cluster-Randomized Trial. Clin Infect Dis (2019) 69(10):1780–8. doi: 10.1093/cid/ciz066 PMC6821165 30689757

[141] Sullender WM Fowler KB Gupta V Krishnan A Ram Purakayastha D Srungaram Vln R. Efficacy of Inactivated Trivalent Influenza Vaccine in Rural India: A 3-Year Cluster-Randomised Controlled Trial. Lancet Glob Health (2019) 7(7):e940–e50. doi: 10.1016/S2214-109X(19)30079-8 PMC7347003 31200893

[142] Heikkinen T Ruuskanen O Waris M Ziegler T Arola M Halonen P. Influenza Vaccination in the Prevention of Acute Otitis Media in Children. Am J Dis Child (1991) 145(4):445–8. doi: 10.1001/archpedi.1991.02160040103017 1849344

[143] Baum U Kulathinal S Auranen K Nohynek H. Effectiveness of 2 Influenza Vaccines in Nationwide Cohorts of Finnish 2-Year-Old Children in the Seasons 2015-2016 Through 2017-2018. Clin Infect Dis (2020) 71(8):e255–e61. doi: 10.1093/cid/ciaa050 31955204

[144] Joshi AY Iyer VN St Sauver JL Jacobson RM Boyce TG. Effectiveness of Inactivated Influenza Vaccine in Children Less Than 5 Years of Age Over Multiple Influenza Seasons: A Case-Control Study. Vaccine (2009) 27(33):4457–61. doi: 10.1016/j.vaccine.2009.05.038 19490957

[145] Heinonen S Silvennoinen H Lehtinen P Vainionpää R Ziegler T Heikkinen T. Effectiveness of Inactivated Influenza Vaccine in Children Aged 9 Months to 3 Years: An Observational Cohort Study. Lancet Infect Dis (2011) 11(1):23–9. doi: 10.1016/S1473-3099(10)70255-3 21106443

[146] Turner N Pierse N Bissielo A Huang QS Baker MG Widdowson MA. The Effectiveness of Seasonal Trivalent Inactivated Influenza Vaccine in Preventing Laboratory Confirmed Influenza Hospitalisations in Auckland, New Zealand in 2012. Vaccine (2014) 32(29):3687–93. doi: 10.1016/j.vaccine.2014.04.013 PMC4620982 24768730

[147] Qin Y Zhang Y Wu P Feng S Zheng J Yang P. Influenza Vaccine Effectiveness in Preventing Hospitalization Among Beijing Residents in China, 2013-15. Vaccine (2016) 34(20):2329–33. doi: 10.1016/j.vaccine.2016.03.068 27026147

[148] Zhang Y Wu P Feng L Yang P Pan Y Feng S. Influenza Vaccine Effectiveness Against Influenza-Associated Hospitalization in 2015/16 Season, Beijing, China. Vaccine (2017) 35(23):3129–34. doi: 10.1016/j.vaccine.2017.03.084 28456530

[149] Chua H Chiu SS Chan ELY Feng S Kwan MYW Wong JSC. Effectiveness of Partial and Full Influenza Vaccination Among Children Aged <9 Years in Hong Kong, 2011-2019. J Infect Dis (2019) 220(10):1568–76. doi: 10.1093/infdis/jiz361 PMC6782104 31290537

[150] Segaloff HE Leventer-Roberts M Riesel D Malosh RE Feldman BS Shemer-Avni Y. Influenza Vaccine Effectiveness Against Hospitalization in Fully and Partially Vaccinated Children in Israel: 2015-2016, 2016-2017, and 2017-2018. Clin Infect Dis (2019) 69(12):2153–61. doi: 10.1093/cid/ciz125 30753347

[151] Sofia Arriola C El Omeiri N Azziz-Baumgartner E Thompson MG Sotomayor-Proschle V Fasce RA. Influenza Vaccine Effectiveness Against Hospitalizations in Children and Older Adults-Data From South America, 2013-2017. A Test Negative Design. Vaccine X (2019) 3:100047. doi: 10.1016/j.jvacx.2019.100047 31867577 PMC6904815

[152] Buchan SA Chung H Campitelli MA Crowcroft NS Gubbay JB Karnauchow T. Vaccine Effectiveness Against Laboratory-Confirmed Influenza Hospitalizations Among Young Children During the 2010-11 to 2013-14 Influenza Seasons in Ontario, Canada. PloS One (2017) 12(11):e0187834. doi: 10.1371/journal.pone.0187834 29149183 PMC5693284

[153] Campbell AP Ogokeh C Lively JY Staat MA Selvarangan R Halasa NB. Vaccine Effectiveness Against Pediatric Influenza Hospitalizations and Emergency Visits. Pediatrics (2020) 146(5):e20201368. doi: 10.1542/peds.2020-1368 33020249

[154] Loeb M Russell ML Moss L Fonseca K Fox J Earn DJ. Effect of Influenza Vaccination of Children on Infection Rates in Hutterite Communities: A Randomized Trial. JAMA (2010) 303(10):943–50. doi: 10.1001/jama.2010.250 20215608

[155] Pepin S Samson SI Alvarez FP Dupuy M Gresset-Bourgeois V De Bruijn I. Impact of a Quadrivalent Inactivated Influenza Vaccine on Influenza-Associated Complications and Health Care Use in Children Aged 6 to 35months: Analysis of Data From a Phase III Trial in the Northern and Southern Hemispheres. Vaccine (2019) 37(13):1885–8. doi: 10.1016/j.vaccine.2019.01.059 30745147

[156] Ruohola A Meurman O Nikkari S Skottman T Salmi A Waris M. Microbiology of Acute Otitis Media in Children With Tympanostomy Tubes: Prevalences of Bacteria and Viruses. Clin Infect Dis (2006) 43(11):1417–22. doi: 10.1086/509332 PMC7107988 17083014

[157] Norhayati MN Ho JJ Azman MY. Influenza Vaccines for Preventing Acute Otitis Media in Infants and Children. Cochrane Database Syst Rev (2017) 10:CD010089. doi: 10.1002/14651858.CD010089.pub3 29039160 PMC6485791

[158] Fink G Orlova-Fink N Schindler T Grisi S Ferrer APS Daubenberger C. Inactivated Trivalent Influenza Vaccination Is Associated With Lower Mortality Among Patients With COVID-19 in Brazil. BMJ Evidence-Based Med (2021) 26:192–3. doi: 10.1136/bmjebm-2020-111549 33310766

[159] Reichert TA Sugaya N Fedson DS Glezen WP Simonsen L Tashiro M. The Japanese Experience With Vaccinating Schoolchildren Against Influenza. N Engl J Med (2001) 344(12):889–96. doi: 10.1056/NEJM200103223441204 11259722

[160] Guthmiller JJ Utset HA Henry C Li L Zheng NY Sun W. An Egg-Derived Sulfated N-Acetyllactosamine Glycan Is an Antigenic Decoy of Influenza Virus Vaccines. mBio (2021) 12(3):e0083821. doi: 10.1128/mBio.00838-21 34126773 PMC8263001

[161] Jung J Mundle ST Ustyugova IV Horton AP Boutz DR Pougatcheva S. Influenza Vaccination in the Elderly Boosts Antibodies Against Conserved Viral Proteins and Egg-Produced Glycans. J Clin Invest (2021) 131(13):e148763. doi: 10.1172/JCI148763 PMC8245176 34196304

[162] Barr IG Donis RO Katz JM McCauley JW Odagiri T Trusheim H. Cell Culture-Derived Influenza Vaccines in the Severe 2017-2018 Epidemic Season: A Step Towards Improved Influenza Vaccine Effectiveness. NPJ Vaccines (2018) 3:44. doi: 10.1038/s41541-018-0079-z 30323955 PMC6177469

[163] DiazGranados CA Denis M Plotkin S. Seasonal Influenza Vaccine Efficacy and Its Determinants in Children and Non-Elderly Adults: A Systematic Review With Meta-Analyses of Controlled Trials. Vaccine (2012) 31(1):49–57. doi: 10.1016/j.vaccine.2012.10.084 23142300

[164] Eun BW Lee TJ Lee J Kim KH Kim DH Jo DS. A Randomized, Double-Blind, Active-Controlled Phase III Trial of a Cell Culture-Derived Quadrivalent Inactivated Influenza Vaccine in Healthy South Korean Children and Adolescents 6 Months to 18 Years of Age. Pediatr Infect Dis J (2019) 38(9):e209–e15. doi: 10.1097/INF.0000000000002406 31335572

[165] Oh CE Choi UY Eun BW Lee TJ Kim KH Kim DH. A Randomized, Double-Blind, Active-Controlled Clinical Trial of a Cell Culture-Derived Inactivated Trivalent Influenza Vaccine (NBP607) in Healthy Children 6 Months Through 18 Years of Age. Pediatr Infect Dis J (2018) 37(6):605–11. doi: 10.1097/INF.0000000000001973 29528914

[166] Diez-Domingo J de Martino M Lopez JG Zuccotti GV Icardi G Villani A. Safety and Tolerability of Cell Culture-Derived and Egg-Derived Trivalent Influenza Vaccines in 3 to <18-Year-Old Children and Adolescents at Risk of Influenza-Related Complications. Int J Infect Dis (2016) 49:171–8. doi: 10.1016/j.ijid.2016.06.018 27343983

[167] Nolan T Chotpitayasunondh T Capeding MR Carson S Senders SD Jaehnig P. Safety and Tolerability of a Cell Culture Derived Trivalent Subunit Inactivated Influenza Vaccine Administered to Healthy Children and Adolescents: A Phase III, Randomized, Multicenter, Observer-Blind Study. Vaccine (2016) 34(2):230–6. doi: 10.1016/j.vaccine.2015.11.040 26643931

[168] Richards KA Moritzky S Shannon I Fitzgerald T Yang H Branche A. Recombinant HA-Based Vaccine Outperforms Split and Subunit Vaccines in Elicitation of Influenza-Specific CD4 T Cells and CD4 T Cell-Dependent Antibody Responses in Humans. NPJ Vaccines (2020) 5(1):77. doi: 10.1038/s41541-020-00227-x PMC7450042 32884842

[169] Chaudhary N Weissman D Whitehead KA. mRNA Vaccines for Infectious Diseases: Principles, Delivery and Clinical Translation. Nat Rev Drug Discovery (2021) 1–22. doi: 10.1038/s41573-021-00283-5 34433919 PMC8386155

[170] Gray KJ Bordt EA Atyeo C Deriso E Akinwunmi B Young N. Coronavirus Disease 2019 Vaccine Response in Pregnant and Lactating Women: A Cohort Study. Am J Obstet Gynecol (2021) 225(3):303.e1–303.e17. doi: 10.1101/2021.03.07.21253094 33775692 PMC7997025

[171] Rottenstreich A Zarbiv G Oiknine-Djian E Zigron R Wolf DG Porat S. Efficient Maternofetal Transplacental Transfer of Anti- SARS-CoV-2 Spike Antibodies After Antenatal SARS-CoV-2 BNT162b2 mRNA Vaccination. Clin Infect Dis (2021), ciab266. doi: doi: 10.1093/cid/ciab266 33822014 PMC8083549

